# Performance Estimation and Ex Vivo Validation of Untethered Magnetic Robots in Soft Tissue

**DOI:** 10.1002/advs.77011

**Published:** 2026-08-14

**Authors:** Leendert‐Jan W. Ligtenberg, Thijs J. van der Burg, Stijn Y. Kolkman, Santiago Saavedra Castano, David Fernandez Rivas, Anke Klingner, Roger M. L. M. Lomme, Lucas S. Joziasse, Dorothee Wasserberg, H. Remco Liefers, Doron Ben Ami, Udi Sadeh, Oded Shoseyov, Giulio Dagnino, Pascal Jonkheijm, Jurgen J. Fütterer, Stefano Stramigioli, J. Frank W. Nijsen, Michiel C. Warlé, Islam S. M. Khalil

**Affiliations:** ^1^ RAM—Robotics and Mechatronics University of Twente Enschede The Netherlands; ^2^ Radboud University Medical Center Nijmegen The Netherlands; ^3^ Technical Medical Centre University of Twente Enschede The Netherlands; ^4^ Mesoscale Chemical Systems, MESA+ Institute University of Twente Enschede The Netherlands; ^5^ Department of Physics German University in Cairo New Cairo Egypt; ^6^ Department of Dentistry‐Regenerative Biomaterials Radboud University Medical Center Nijmegen The Netherlands; ^7^ LipoCoat B.V. Enschede The Netherlands; ^8^ Department of Molecules and Materials, Biointerface Chemistry Lab University of Twente Enschede The Netherlands; ^9^ Triticum Medical Ramat HaSharon Israel; ^10^ The Hebrew University of Jerusalem Rehovot Israel; ^11^ Department of Surgery, Division of Vascular and Transplant Surgery Radboud University Medical Center Nijmegen Nijmegen The Netherlands

**Keywords:** brain tissue, magnetic robots, step‐out frequency, viscoelasticity

## Abstract

The navigation and control of untethered magnetic robots (UMRs) within soft brain tissue critically depend on their ability to maintain synchronized rotation under applied rotational magnetic fields. The fundamental upper limit of this magnetic synchronization, known as the step‐out frequency, directly affects propulsion efficiency and control stability. This study presents an empirical model for predicting the step‐out frequency of UMRs operating in ex vivo brain tissue with viscoelastic properties representative of the in vivo environment. Using Buckingham Pi dimensional analysis, we derive a compact set of dimensionless groups that capture the effects of robot geometry and tissue viscoelasticity, with magnetic torque used as the scaling basis. Experimental validation with spiral‐type UMRs in gelatin‐based phantoms shows that the step‐out frequency decreases exponentially, from approximately 30 Hz in low‐stiffness media (complex shear modulus ∼400 Pa) to below 1 Hz in high‐stiffness media (complex shear modulus ∼1600 Pa). The found relationship allows for rapid estimation of robot performance limits using a single calibration point, enabling efficient evaluation of robot design and actuation parameters. The resulting framework supports the clinical translation of magnetic microrobots for precise therapeutic and surgical interventions in brain tissue.

## Graphical abstract

This study presents an empirical model that quantitatively predicts the step‐out frequency of screw‐type untethered magnetic robots (UMRs) in soft viscoelastic environments, including ex vivo brain tissue. By integrating key parameters into a dimensionless framework, the model enables estimation of robot performance across a range of biological conditions. This approach supports systematic UMR design optimization for minimally invasive neurosurgical interventions.

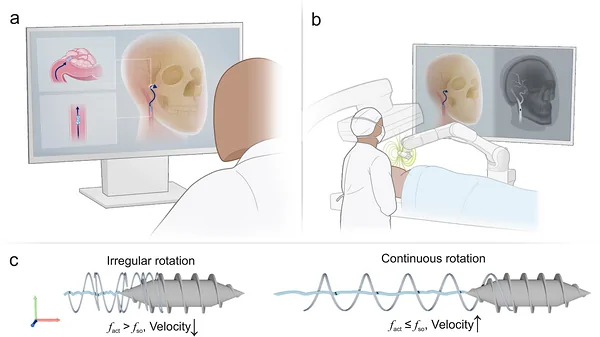

## 1 Introduction

Magnetically actuated untethered robots spanning the micro‐, sub‐millimeter‐, and millimeter‐scale are emerging as powerful tools for minimally invasive medical interventions [1, 2], particularly in the context of navigating the intricate and fragile anatomy of the living human body (Figure 1). Conditions such as stroke, tumors, and vascular malformations demand highly precise instruments capable of traversing tortuous cerebral pathways without causing iatrogenic injury. Untethered magnetic robots (UMRs) address this challenge by using externally applied magnetic fields for wireless propulsion and steering, enabling highly maneuverable, targeted access to deep‐seated regions [3].

**FIGURE 1:**
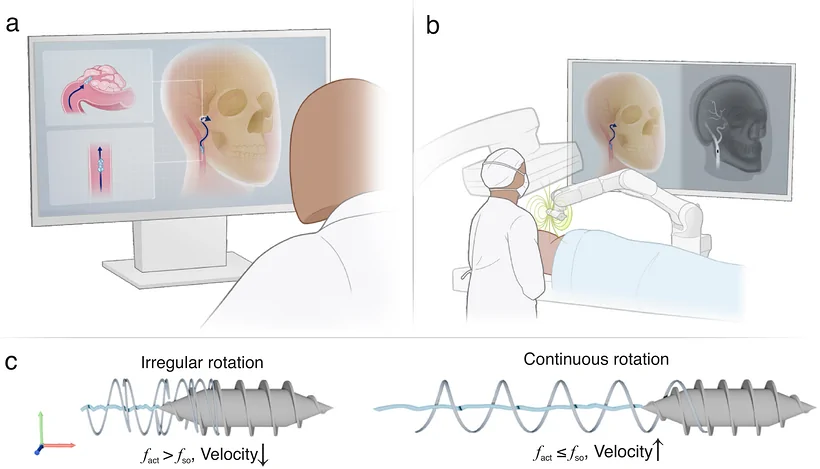
Concept and magnetic actuation of an untethered magnetic robot (UMR) for targeted navigation and drilling in brain tissue. (a) The schematic illustrates a UMR navigating through the cerebral vasculature toward a deep‐seated lesion. Upon reaching the target site, the UMR is capable of penetrating the arterial wall and advancing into the brain parenchyma to access regions that are otherwise inaccessible via conventional techniques. (b) Navigation and propulsion are achieved through a robotically controlled rotating permanent magnet, guided in real‐time by a C‐arm imaging system. (c) The step‐out frequency ($f_{\mathrm{so}}$) represents the critical threshold between low‐frequency actuation ($f_{\mathrm{act}}$), where the UMR remains magnetically synchronized and follows the rotating field (right), and high‐frequency actuation, where synchronization fails and magnetic coupling is lost, resulting in minimal to no motion within the same time frame (left).

The small size of microrobots, in combination with their wireless actuation, could revolutionize medical interventions. Their wide range of possible geometries and well defined control strategies [4] enable various functionalities. Recent studies have shown the potential of UMRs for medical applications such as, radiotherapy [5, 6], drug delivery [3, 7, 8, 9], tissue marking [10, 11], biosensing [12, 13], tissue ablation [14, 15], and more [16]. Therefore, UMRs offer to bring a promising medical platform, which overcomes the current limitations of targeted medicine within intricate organs, such as the brain [17]. The development of such a medical platform requires an adaptable framework to support the evaluation of UMR efficiency. While UMRs can be efficient in a biological medium or tissue, they should also be precisely controllable within the complex interplay between physiological tissues. UMRs that would not fulfill this requirement need to be excluded from consideration for a medical platform [18].

While advances in control, imaging, and actuation have significantly improved UMR navigation in fluids [19, 20, 21], a critical barrier remains in understanding and predicting their behavior in viscoelastic tissues. Biological tissues such as the brain are not Newtonian fluids but viscoelastic media characterized by complex shear moduli that resist motion in nonlinear ways [22]. This medium characteristic causes a particular limiting factor: the “step‐out frequency”, the maximum actuation frequency at which a UMR can maintain synchronous rotation with an external magnetic field. Beyond this threshold, the UMR loses synchrony, drastically reducing its propulsion efficiency and jeopardizing navigation precision. Despite its clinical relevance, current literature lacks predictive models that account for the combined effects of magnetic torque, geometric design, and viscoelastic properties on the step‐out frequency.

Previous studies on magnetic microrobot locomotion have predominantly focused on motion through idealized Newtonian fluids, often relying on numerical simulations or simplified analytical models. Sendoh et al. have conducted early experimental demonstrations of screw‐shaped UMRs actuated by rotating permanent magnets, successfully achieving propulsion through biological tissues, such as muscle and liver [23]. Their findings validated the feasibility of rotational magnetic actuation in dense, viscoelastic environments and highlighted the importance of helical geometry in overcoming tissue resistance. Mahoney et al. have revealed that screw‐based devices operating in tissue are subject to competing stabilizing and destabilizing magnetic torques during steering [22]. Notably, they have shown that above the step‐out threshold, destabilizing torques dominate, resulting in a loss of directional control and trajectory instability. This work showed the need to account for dynamic magnetic interactions in soft media when designing robust control strategies. Building on this, Nelson et al. have developed an empirical framework to predict the turning radius of magnetically actuated screws in soft tissue, integrating magnetic, geometric, and tissue properties through dimensional analysis [24]. Their findings demonstrated that turning behavior can be captured using a limited set of dimensionless parameters, providing an important foundation for predictive modeling in viscoelastic environments.

Complementary studies have further highlighted the role of geometry of microrobots and tissue‐specific resistance in microrobot performance. Li et al. have demonstrated that twisted ribbon microrobots with high aspect‐ratio cross‐sections and chemically etched tips can effectively propel through dense, viscoelastic tissues such as liver tissue [25]. Their results emphasized the importance of structural design and localized stress concentration for overcoming mechanical resistance in biological substrates. Xiang et al. have designed an impact‐driven magnetic robot using reciprocal oscillations to reduce the resistance from the environment [26]. Their experiments showed successful navigation in both liquid and granular media, emphasizing the benefit of using oscillations to reduce tissue resistance. In addition, Ullrich et al. have validated the feasibility of precise magnetic manipulation of microrobots within the vitreous body of rabbit eyes, illustrating both translational and rotational mobility in low‐viscosity and viscoelastic intraocular environments [27]. Their study highlighted the significant impact of viscoelastic resistance, particularly due to collagen fiber entanglement, and introduced practical electromagnetic actuation strategies, such as the OctoMag system [28], for in vivo ophthalmic applications under wireless control.

Despite these contributions, a generalizable and experimentally validated predictive framework for microrobot performance in real biological tissues with viscoelastic characteristics remains lacking. Most existing models are limited in scope, either omitting frequency‐dependent synchronization limits or failing to validate performance in physical phantoms with tunable mechanical properties that accurately reflect biological tissue behavior. The development of such a framework would accelerate the bench‐to‐bedside translation of UMRs and reduce the need for animal tissue. Furthermore, it would provide clinicians with a quantitative tool for selecting the most appropriate UMR design and magnetic actuation strategy for a given clinical procedure based on the mechanical properties of the target tissue and the requirements of the intended navigation task. This would enable an application‐specific design strategy, which is essential for the successful execution of a well‐defined medical project from start to finish [29]. This study lays the foundation for such a framework by developing and experimentally validating an empirical model that predicts the step‐out frequency of UMRs in soft biological tissues, such as brain tissue. The model captures the nonlinear relationship between step‐out frequency and tissue stiffness and provides a framework for anticipating microrobot performance under varying mechanical conditions.

The modeling framework is based on dimensional analysis using the Buckingham Pi theorem [30], incorporating magnetic actuation parameters, robot geometry, and the viscoelastic modulus of the surrounding medium. A single spiral‐type UMR design, often used in soft tissues [22, 23, 24], was used to establish the empirical relationship between the derived Pi groups and the step‐out frequency, forming the predictive model. Three additional UMR designs, each with distinct geometric and magnetic properties (Figure 2), were subsequently used to validate the model across a broad range of mechanical environments. Magnetic actuation was achieved using a rotating permanent magnet operating in the low‐millitesla range, enabling wireless propulsion and steering. The rheological properties of both phantom and brain tissues were characterized to provide accurate mechanical inputs for model calibration and validation.

**FIGURE 2:**
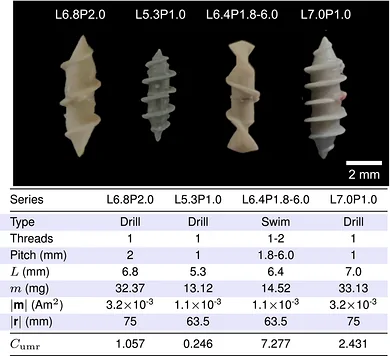
Overview of the four untethered magnetic robot (UMR) designs evaluated in this study. Each UMR varies in length, pitch, and magnetic moment to assess the influence of geometric and magnetic parameters on step‐out behavior in viscoelastic media. The drill‐type UMRs have a single thread with a constant pitch, while UMR‐L6.4P1.8‐6.0 is a smooth‐bodied UMR with varying pitch designed for swimming. L denotes the length of the UMR, m is its mass, |m| is the magnitude of its magnetic moment, and |r| represents the distance between the actuator's rotating permanent magnet and the UMR. The constant $C_{\mathrm{umr}}$ is a calibration factor dependent on the UMR geometry and the applied magnetic field strength, determined empirically at a gelatin concentration of 4 wt.%, for each UMR.

Validation is performed through testing in gelatin‐based tissue phantoms and perfused *ex vivo* ovine brain models. The results demonstrate that step‐out frequency decays exponentially with increasing tissue stiffness, and the resulting dimensionless formulation enables estimation of operational limits for new UMR designs from a single calibration point. This significantly reduces experimental workload and advances the translational feasibility of microrobotic applications in soft tissues, including interventions in neurosurgery.

## 2 Results and Discussion

### 2.1 Locomotion of Magnetic Robots Inside Ex Vivo Cerebral Tissue

To assess the ability of UMRs to move through real brain tissue, locomotion experiments were performed using a single drill‐type design with a length of L=7.0 mm and a thread pitch of 1.0 mm (UMR‐L7.0P1.0) in perfused ex vivo ovine brain samples (Materials and Methods). The UMR contains a ferromagnetic core with a transverse magnetic moment, |m|, enabling it to rotate about its longitudinal axis and advance forward in a screw‐like fashion under the influence of an externally applied rotating magnetic field. To ensure compatibility with biological environments, the UMR is coated with a lipid‐based biocompatible layer that promotes hemocompatibility and minimizes adverse interactions with tissue and blood [20, 31] (Materials and Methods). The UMR was inserted through an incision (Figure 3a) and actuated using rotating magnetic fields generated by a permanent magnet mounted on a robotic arm, where the distance between the magnet and the UMR, defined by the variable |r|, was used to control the strength of the magnetic field experienced by the UMR.

**FIGURE 3:**
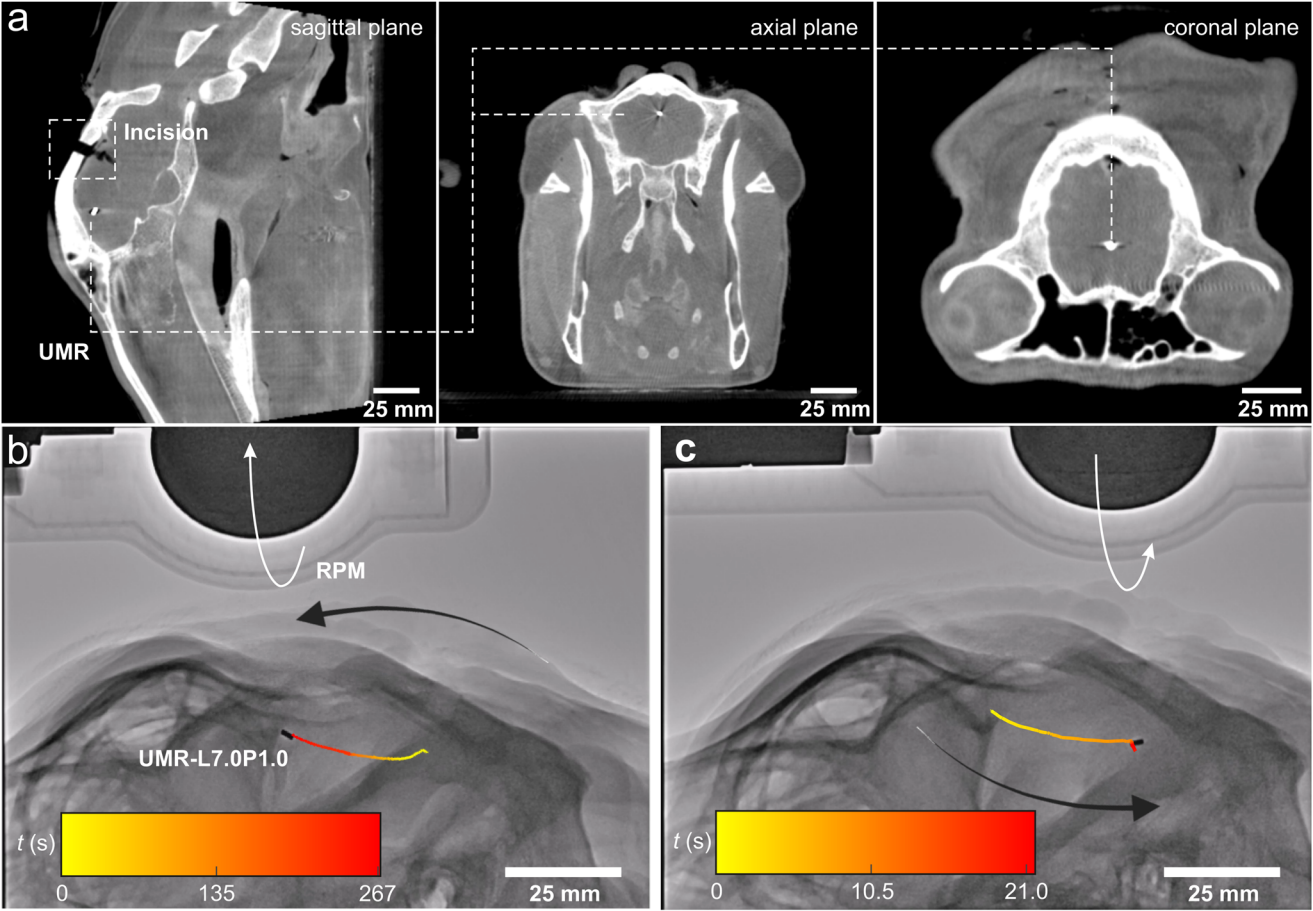
A drill‐type untethered magnetic robot (UMR) with a length of 7.0 mm and thread pitch of 1.0 mm (UMR‐L7.0P1.0) is introduced through an incision and propelled using externally applied rotating magnetic fields. (a) Sagittal, axial, and coronal planes showing the position of the UMR within the brain tissue, with the sagittal view showing the incision used for insertion. (b) A rotating magnetic field, generated by a robotically actuated permanent magnet (RPM), induces wireless propulsion of the UMR through brain tissue via helical motion (Movie S1). (c) Reversing the direction of magnetic field rotation enables controlled backward locomotion, allowing the UMR to retrace its original path toward the insertion site (Movie S2).

The spatial relationship between the insertion point and the UMRs final position was visualized using Cone Beam Computed Tomography (CBCT). The sagittal plane of the CBCT scan reveals the incision, shown by a white dashed box, Figure 3a, through which the UMR enters the brain parenchyma. The UMRs position is also distinctly indicated in the axial and coronal planes, allowing for multi‐planar confirmation of its depth, trajectory, and orientation within the cerebral architecture. While the current experimental setup introduces the UMR through an incision for visualization and validation purposes, the envisioned clinical pathway involves endovascular access [14]. In future applications, the UMRs will be designed to be delivered via the vascular system, where they can swim through the bloodstream, drill through the arterial wall at targeted locations, and penetrate the surrounding brain tissue to reach deep‐seated lesions without the need for open surgical access.

Under low‐frequency magnetic actuation (1.8 Hz to 3.0 Hz), UMR‐L7.0P1.0 demonstrated continuous helical propulsion through soft cortical tissue. Forward locomotion (Figure 3b) exhibited greater resistance compared to backward motion (Figure 3c), as indicated by the higher translational speed observed when the UMR was actuated in reverse. The velocity increased from 0.2 mm/s during forward locomotion (Movie S1) to 2.9 mm/s during backward locomotion (Movie S2). This substantial increase suggests that the UMR created a low‐resistance path while drilling through the tissue, which it subsequently retraced during backward navigation toward the insertion site, thereby encountering significantly reduced mechanical resistance.

During locomotion and targeting of diseased cells within brain tissue, precise steering of the UMR toward specific anatomical targets is essential. This can be achieved using externally applied magnetic fields, guided by real‐time feedback from X‐ray Fluoroscopy images acquired with a C‐arm imaging system (Figure 1). In such procedures, both the translational and angular orientation of the UMR must be actively controlled, requiring simultaneous spinning about its longer axis and steering toward the desired trajectory. The rotating magnetic field not only induces helical propulsion but also applies a torque component about the vertical axis of the UMRs body‐fixed frame, enabling controlled directional changes [22]. This capability was demonstrated in a low‐stiffness gelatin‐based phantom, where the UMR was controlled in closed‐loop toward multiple targets, as shown in Figure 4.

**FIGURE 4:**
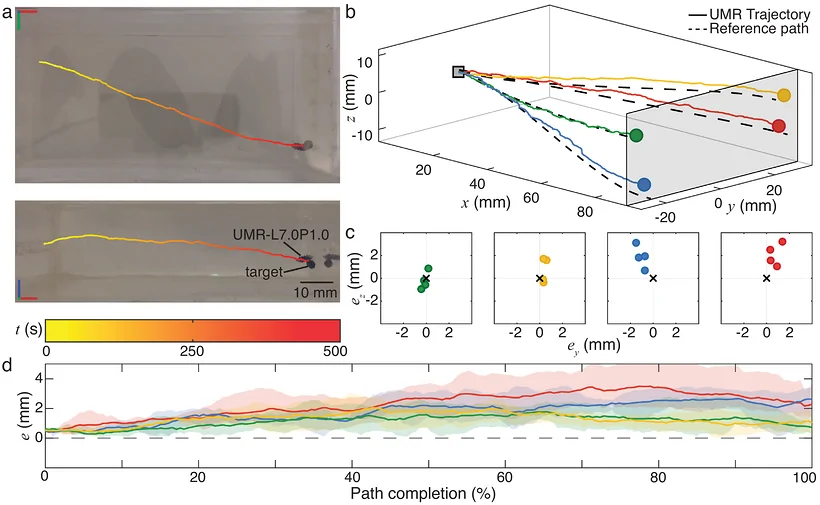
Closed‐loop control of an untethered magnetic robot (UMR) UMR‐L7.0P1.0 in a 4 wt.% gelatin phantom. (a) The UMR was steered from its insertion (left) toward its target (right), the target was reached after 500 seconds (Movie S3). (b) Four different reference trajectories and the corresponding UMR trajectories, the UMR was able to navigate along all trajectories. (c) Final positions of the UMR with respect to the target positions, all targeting experiments were repeated 4 times. (d) Closed‐loop navigation error of the UMR along each trajectory, the solid curve represents the mean error of the UMR, while the shaded region represents the standard deviation.

### 2.2 Closed‐Loop Control of Untethered Magnetic Robots

The efficacy of using UMRs within brain tissue is strongly connected to their control accuracy. The UMR must be capable of navigating around critical areas while still reaching its target within acceptable error margins. To demonstrate the UMR control strategy, UMR‐L7.0P1.0 was controlled in closed‐loop within a 4 weight percent (wt.%) gelatin phantom (Movie S3). By using a dual‐camera setup the UMR position and orientation can be reconstructed in real‐time, allowing for a closed‐loop control system (Materials and Methods). The direction of the rotating magnetic field serves as the input, dictating the UMR's orientation and thus its propulsion vector. Yaw control was implemented by varying the RPM yaw angle, exploiting the UMR's natural alignment with the field. Pitch control was achieved by introducing a controlled lateral offset between the UMR and the RPM, positioning the RPM behind the UMR will result in an upward pitch of the UMR and vice versa.

Figure 4a depicts a representative UMR trajectory as it navigates from its starting point (left) toward its target (right). Despite initial misalignment the UMR successfully oriented itself toward the target. As the UMR approached its target, yaw adjustments allowed the UMR to reach the target perpendicular to the phantom walls (Movie S3). Figure 4b shows four UMR reference paths and corresponding UMR trajectories for each path. Throughout navigation within the phantom, the UMR maintained close proximity to its designated path, despite occasional deviations. Critically, the closed‐loop control system consistently corrected these deviations, guiding the UMR back toward its reference path. Targeting accuracy, presented in Figure 4(c), shows the UMR achieved sub‐millimeter errors (0.70 ± 0.34 mm and 1.06 ± 0.76 mm for green/yellow) for some paths. Though larger errors (2.19 ± 1.01 mm and 2.25 ± 0.97 mm for blue/red) occurred, primarily due to increased vertical deviation, indicating that magnetic attraction forces dominated over gravitational forces. The error between the UMR to its reference path is shown in Figure 4d, revealing that while errors reached up to 2 mm, for the green and yellow trajectories this error was corrected back down to around 1 mm, whereas deviations remained elevated for the blue and red paths.

Closed‐loop control represents a crucial advancement for UMRs toward clinical translation in soft tissue environments. However, the step‐out frequency is a key factor in control stability [22]. Consequently, the closed‐loop controller should be carefully tuned for each UMR and its corresponding actuator speed. Unless equipped with a method to determine the relationship between tissue stiffness and step‐out frequency, it may be unavoidable for the UMR to become unstable and fall out of sync with the actuating field. Once the actuation frequency exceeds the step‐out frequency, the UMR can no longer maintain synchronous rotation with the applied magnetic field. This occurs when the magnetic torque becomes insufficient to overcome the mechanical resistance of the surrounding tissue, resulting in a significant reduction or complete loss of translational motion. Under these conditions, the UMR cannot reliably respond to changes in the actuation field, making directional control, steering, and navigation impossible (Movie S4). Therefore, the step‐out frequency must be characterized and accounted for in the control strategy.

### 2.3 Characterization of Step‐Out Frequency in Viscoelastic Media

To quantify how viscoelastic tissue properties affect the locomotion performance of UMRs, measurements of the step‐out frequency were performed using four UMR designs (Figure 2) in gelatin‐based phantoms with tunable mechanical properties. To characterize the viscoelastic properties of the gelatin‐based phantoms, frequency‐sweep rheological tests were conducted on samples with concentrations ranging from 3 wt.% to 7 wt.%. The storage modulus (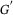), loss modulus (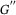), and complex modulus ($|G^{*}|$) were measured across all concentrations (Figure 5a–c). The storage modulus 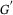 exhibited minimal frequency dependence, indicating the formation of a stable, well‐structured gel network. As gelatin concentration increased, 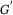 also increased, reflecting greater stiffness and enhanced elastic response of the phantom material. Although 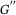 increases with gelatin concentration, it remains nearly two orders of magnitude lower than 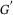, indicating that the phantom material exhibits predominantly elastic behavior with minimal viscous dissipation. Due to the relatively small contribution of 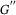, the complex modulus $|G^{*}|$ is primarily governed by the storage modulus and thus also shows limited frequency dependence.

**FIGURE 5:**
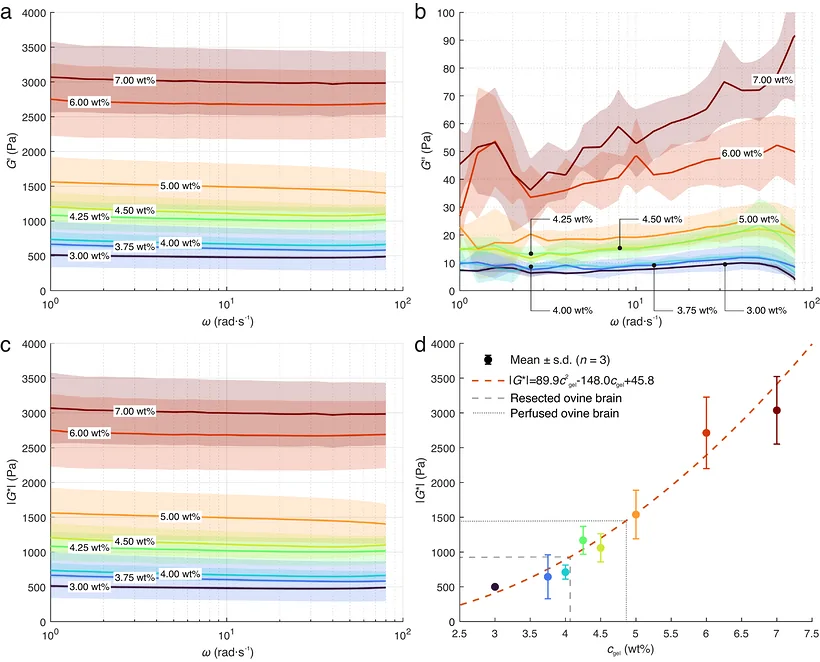
Rheological characterization of gelatin‐based phantoms with concentrations ranging from 3 wt.% to 7 wt.%. The storage modulus (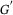), loss modulus (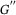), and complex modulus ($|G^{*}|$) are measured to quantify the viscoelastic properties of the medium as a function of gelatin concentration. (a) 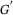 increases with gelatin concentration, indicating that the elastic (solid‐like) behavior of the phantom becomes more dominant as the gelatin concentration increases. (b) 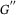 increases with gelatin concentration, reflecting an increase in the viscous (liquid‐like) component of the phantom's mechanical response as the material becomes denser. (c) $|G^{*}|$, representing the overall resistance to deformation, increases with gelatin concentration, capturing the combined effects of both elastic and viscous contributions to the phantom's viscoelastic behavior. (d) Empirical relation between gelatin concentration $c_{\mathrm{gel}}$ (wt.%) and $|G^{*}|$ (at ω≈ 2 $\mathrm{rad}\cdot s^{-1}$) of the phantom material. The theoretical prediction closely matches the experimental measurements, confirming its validity for estimating the viscoelastic properties of gelatin‐based media across varying stiffness levels. The gray dashed lines represent the estimated complex moduli and corresponding gelatin concentrations of resected and perfused ovine brain tissue.

The measured values of $|G^{*}|$ at approximately 2 Hz are compared against the empirical relation reported by Chen [32], as shown in Figure 5d. The relationship between gelatin concentration and complex shear modulus, validated over the range of 3 wt.% to 7 wt.% (Materials and Methods), is described by the empirical equation [32, 33]:

(1) $$|G^{*}|=89.9c_{\mathrm{gel}}^{2}-148.0c_{\mathrm{gel}}+45.8\;,$$

where $c_{\mathrm{gel}}$ is the gelatin concentration in weight percent. The measured data in this study show strong agreement with this empirical model, Figure 5d. Deviations are attributed to experimental variations, such as slight differences in ambient temperature and sample thickness during rheological testing.

Step‐out frequency experiments on the four UMR designs show an exponential decay in step‐out frequency as a function of the medium's complex shear modulus $|G^{*}|$, Figure 6. UMR‐L6.8P2.0, equipped with a large magnetic moment ($3.2\times 10^{-3}$ $A\cdot m^{2}$), was tested across media ranging from 3.75 wt.% to 4.5 wt.% gelatin concentration, corresponding to $|G^{*}|$ values from approximately 750 Pa to 1200 Pa. The observed step‐out frequencies ranged from 5.38±2.41 Hz in the softest medium to 0.56 ±0.12 Hz in the stiffest, confirming an exponential relation between stiffness and step‐out frequency (Movie S4).

**FIGURE 6:**
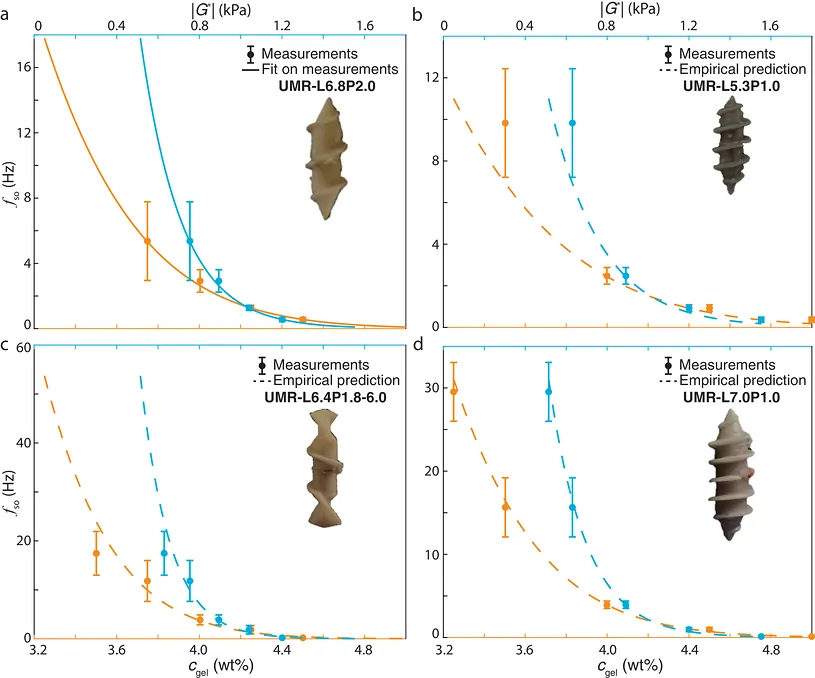
Step‐out frequency, $f_{\mathrm{so}}$, characterization of four screw‐type untethered magnetic robots (UMRs) in viscoelastic media of varying stiffness. Medium stiffness is modulated by altering the gelatin concentration, $c_{\mathrm{gel}}$, and the complex shear modulus of each sample is calculated using the empirical relation $|G^{*}|=89.9c_{\mathrm{gel}}^{2}-148.0c_{\mathrm{gel}}+45.8$. This enables systematic evaluation of how viscoelastic resistance influences the synchronization limit of each UMR design. The drill‐type robots include UMR‐L6.8P2.0, UMR‐L5.3P1.0, and UMR‐L7.0P1.0, while UMR‐L6.4P1.8‐6.0 is a smooth‐bodied swimming variant with no continuous helical threads. (a) UMR‐L6.8P2.0 exhibits an exponential decrease in step‐out frequency, from $f_{\mathrm{so}}=5.3$ Hz in a low‐stiffness medium with $|G^{*}|=750$ Pa to 0.56 Hz at $|G^{*}|=1200$ Pa (Movie S4). (b) UMR‐L5.3P1.0 exhibits an exponential decrease in step‐out frequency, from $f_{\mathrm{so}}=9.8$ Hz in a low‐stiffness medium with $|G^{*}|=600$ Pa to 0.37 Hz at $|G^{*}|=1600$ Pa (Movie S5). (c) UMR‐L6.4P1.8‐6.0 exhibits an exponential decrease in step‐out frequency, from $f_{\mathrm{so}}=17.4$ Hz in a low‐stiffness medium with $|G^{*}|=600$ Pa to 0.22 Hz at $|G^{*}|=1200$ Pa (Movie S6). (d) UMR‐L7.0P1.0 exhibits an exponential decrease in step‐out frequency, from $f_{\mathrm{so}}=29.5$ Hz in a low‐stiffness medium with $|G^{*}|=500$ Pa to 0.12 Hz at $|G^{*}|=1600$ Pa (Movie S7).

Other UMRs, including UMR‐L5.3P1.0, UMR‐L6.4P1.8–6.0 (a smooth‐bodied swimmer with variable pitch), and UMR‐L7.0P1.0, exhibited performance trends consistent with variations in magnetic moment, geometry, and thread pitch, as shown in Figure 6b–d (Movies S5–S7). For instance, UMR‐L7.0P1.0 maintained higher step‐out frequencies than UMR‐L5.3P1.0 at equivalent medium stiffness. Despite the larger size of UMR‐L7.0P1.0 compared to UMR‐L5.3P1.0, its higher magnetic moment ($3.2\times 10^{-3}A$ ·m 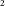 compared to $1.1\times 10^{-3}A$ ·m 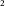) resulted in higher step‐out frequencies at lower complex moduli. As the complex modulus increases, $|G^{*}|$ > 800, UMR‐L5.3P1.0 achieves higher step‐out frequencies. This suggests that as a tissue gets more stiff, a smaller size UMR is preferred over a bigger magnetic torque.

Another interesting observation that can be made is the effect of helical pitch on the step‐out. When we compare UMR‐L7.0P1.0 to UMR‐L6.8P2.0, we can see that UMR‐L7.0P1.0 has a higher step‐out frequency for all tested gelatin concentrations. This can be attributed to the higher drag experienced by the larger pitch. In general, UMRs with larger pitch are designed to travel more per revolution, but thereby experiences more drag torque per revolution, resulting in the lower step‐out frequencies.

UMR‐L6.4P1.8–6.0, lacks continuous helical threading and features a variable pitch design, exhibited a relatively high step‐out frequency for low gelatin concentrations compared to the other robots, likely due to the smaller fin width. However, for higher gelatin concentrations the step‐out frequency drops faster compared to the other designs, Figure 6c. This can be attributed to the variable pitch, where in stiffer media the interplay between the two pitches results in a lower step‐out frequency. The difference in performance underscores the importance of thread geometry in enabling effective torque transmission through viscoelastic environments. Furthermore, this result shows us that the drag torque of this design is higher, meaning in low viscous environments the UMR is able to overcome this higher drag torque and thereby achieving higher propositional speeds. Therefore, this design may offer advantages for navigating low complex modulus media such as non‐Newtonian bodily fluids and reaching target regions in soft tissue via minimally invasive, endovascular pathways [19, 34]. As shown in Figure 6c, its step‐out frequency drops to 0.22±0.08 Hz in a medium with a complex shear modulus of 1200 Pa. These experimental results demonstrate that step‐out frequency is governed by a complex interplay between magnetic actuation strength, robot geometry, and tissue viscoelasticity. While the trends are consistent across different UMR designs and media, a predictive model is needed to unify these dependencies.

### 2.4 Combined Influence of Magnetic Actuation, Robot Geometry, and Tissue Viscoelasticity on the Step‐Out Frequency

To quantify the interplay between magnetic actuation, robot geometry, and tissue viscoelasticity on UMR performance, the experimental results were reformulated using dimensionless analysis based on the Buckingham Pi theorem. Two key dimensionless parameters were derived to capture the governing physical forces. The first, $\Pi_{1}$, is a normalized step‐out frequency that compares the characteristic timescale of magnetic actuation to that of the UMR's inertial response, encompassing how efficiently the magnetic torque drives rotation against resistive forces. The second, $\Pi_{2}$, represents the ratio of the tissue's viscoelastic resistance to the applied magnetic torque, and quantifies how strongly the mechanical properties of the environment influence locomotion. These parameters are defined as:

(2) $$\Pi_{1}=f_{\mathrm{so}}\sqrt{\frac{L^{2}m}{\left|m||B\right|}}\;,\;\Pi_{2}=\frac{|G^{*}|L^{3}}{\left|m||B\right|}\;,$$

where L and m are the length and mass of the UMR, |m| is the magnetic dipole moment, and |B| is the magnetic field strength. This formulation enables generalization across different UMR geometries (Figure 2) and media by collapsing diverse experimental data into a unified model space. Other parameters, such as surface area, thread pitch, thread depth, and other geometric descriptors, could also be incorporated into the dimensionless groups. However, these parameters are often difficult to measure directly, may vary along the length of a single UMR, or can deviate from their nominal design values due to fabrication tolerances and manufacturing imperfections. In addition, some geometric features are highly design‐specific, limiting their applicability across different UMR architectures. In contrast, the UMR length, L, is a well‐defined, robust, and readily measurable quantity that captures the dominant geometric scaling of the robot while preserving model simplicity. By restricting the geometric characterization to L, the proposed framework relies exclusively on parameters that can be measured using basic laboratory equipment, thereby reducing characterization effort, improving reproducibility, and enabling rapid performance estimation across a wide range of UMR designs and tissue environments.

The experimental data (Figure 6) revealed that the dimensionless parameter $\Pi_{1}$, which represents the normalized step‐out frequency, exhibits an exponential decay with increasing $\Pi_{2}$, the dimensionless representation of medium stiffness. This trend reflects the influence of viscoelastic resistance in limiting magnetic torque transmission as the surrounding tissue becomes stiffer. The relationship can be generalized by the following empirical expression:

(3) $$\Pi_{1}=C_{\mathrm{umr}}\exp\left(-1.201\Pi_{2}\right)\;,$$

where $C_{\mathrm{umr}}$ is a calibration constant that incorporates the effects of UMR geometry and magnetic properties. The addition of this calibration constant greatly reduces the search space of possible Pi groups, as it captures the effects of all other parameters that could otherwise form additional Pi groups. The parameter $C_{\mathrm{umr}}$ captures the effects of structural parameters such as the thread geometry and pitch of the screw, as well as material properties such as surface adhesion and surface roughness, in addition to manufacturing tolerances. The decay coefficient 1.201 was determined by fitting the data obtained for UMR‐L6.8P2.0, as shown in Figure 6a using the blue and red solid curves.

Different UMR designs exhibited consistent exponential decay trends in their dimensionless step‐out behavior, but with distinct $C_{\mathrm{umr}}$ values, highlighting the influence of magnetic moment, geometry, and thread pitch on torque transmission and synchronization thresholds. Calibration constants were determined for each UMR design using a single step‐out frequency measurement in a 4% gelatin phantom with known viscoelastic properties. The resulting values were $C_{\mathrm{umr}}=2.430$ for UMR‐L7.0P1.0, $C_{\mathrm{umr}}=0.246$ for UMR‐L5.3P1.0, $C_{\mathrm{umr}}=7.277$ for the smooth‐bodied swimmer UMR‐L6.4P1.8–6.0, and $C_{\mathrm{umr}}=1.057$ for UMR‐L6.8P2.0.

When plotted on a logarithmic scale (Figure 7), the relationship between $\log(\Pi_{1})$ and $\Pi_{2}$ can be given by:

(4) $$\log\left(\Pi_{1}\right)=-1.201\log\left(e\right)\Pi_{2}+\log\left(C_{\mathrm{umr}}\right).$$

Note that log(e) refers to the logarithm with base 10 of the mathematical constant e, yielding a slope of −1.201log(e). Using the relation between $\log(\Pi_{1})$ and $\Pi_{2}$ in Equation (4), allows us to determine the sensitivity S of the step‐out frequency on a logarithmic scale to the magnitude of the complex modulus:

(5) $$S=-1.201\log\left(e\right)\frac{L^{3}}{\left|m||B\right|}.$$

This relation teaches us that a smaller sensitivity can be achieved by lowering the UMR length or increasing the magnetic torque exerted on the UMR.

**FIGURE 7:**
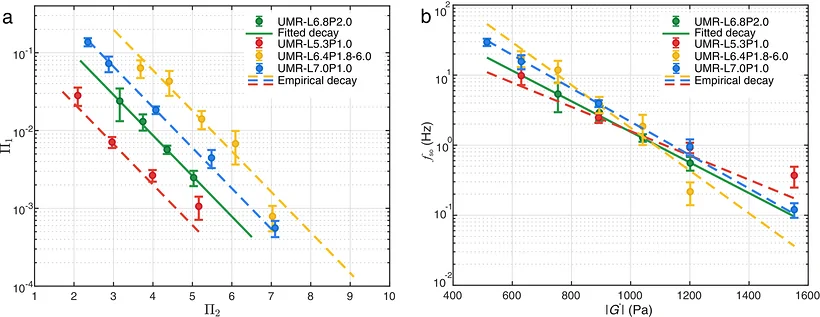
Exponentiation relationship in the dimensionless parameter space and the corresponding actuation parameter space. (a) Dimensionless relationship between normalized step‐out frequency ($\Pi_{1}$) and viscoelastic resistance ($\Pi_{2}$) for four untethered magnetic robot (UMR) designs. Data from experimental measurements collapse onto a single exponential decay trend, confirming that step‐out behavior is predominantly governed by medium stiffness and robot‐specific magnetic and geometric properties. The empirical fit $\Pi_{1}=C_{\mathrm{umr}}\exp\left(-1.201\Pi_{2}\right)$ provides a unified predictive framework across different designs and environments. (b) Relationship of the step‐out frequency and complex modulus of the four UMR designs in gelatin phantoms. The higher step‐out frequency of the smaller UMRs at high complex moduli show that smaller size is preferred over magnetic moment when drilling in tissue.

Using the relation found in Equation (3) allows us to predict the step‐out frequency of any UMR with a known $C_{\mathrm{umr}}$:

(6) $$f_{\mathrm{so}}=\sqrt{\frac{\left|m||B\right|}{L^{2}m}}C_{\mathrm{umr}}\exp\left(-1.201|G^{*}|\frac{L^{3}}{\left|m||B\right|}\right)\;.$$

This equation quantifies the UMR's locomotion limits, incorporating key physical parameters. The prefactor $\sqrt{\left|m||B\right|/\left(L^{2}m\right)}$ establishes a characteristic frequency scale depending on the magnetic field strength, UMR's magnetic moment m, its length L, and mass m. Increased values of |m| or |B| enhance the magnetic driving torque, while greater L or m increase the rotational drag. The exponential term $\exp\left(-1.201|G|L^{3}/\left(|m||B|\right)\right)$ captures the suppressive effect of tissue properties, parameterized by the complex shear modulus $|G^{*}|$, which integrates the medium's elasticity and viscosity.

Altogether, Equation 6 provides a robust and compact predictive framework for estimating the step‐out thresholds of UMRs across a range of viscoelastic environments. It enables rapid evaluation of new UMR designs and magnetic actuation strategies to ensure consistent and reliable performance in soft biological tissues. The empirical prediction of the step‐out frequency using Equation (6) is visualized by the black and red dashed curves in Figure 6b‐d.

For UMR‐L5.3P1.0, the step‐out frequency was predicted as a function of increasing gelatin concentration, $c_{\mathrm{gel}}$, and the corresponding complex shear modulus, $|G^{*}|$. Across most of the tested conditions, the theoretical predictions of Equation (6) closely match the experimentally measured values. However, a noticeable deviation is observed at $|G^{*}|=630$ Pa, where the measured step‐out frequency exceeds the predicted value. This discrepancy is likely caused by accumulated errors in the input parameters, particularly due to the strong temperature dependence of the gelatin phantom properties [35]. Resulting in a mean absolute percentage error (MAPE) of the prediction is 26.7%.

Despite its last measurement point, the empirical predictions for the swimming‐type UMR‐L6.4P18‐6.0 align well with experimental measurements across all other tested gelatin concentrations and their corresponding complex shear moduli (MAPE of 36.7%), as shown in Figure 6c. This consistency suggests that the created empirical model is applicable to multiple UMR sizes and design philosophies.

The empirical model demonstrates good accuracy for the swimming‐type UMR‐L6.4P18‐6.0, with experimental measurements aligning well across all other tested gelatin concentrations except for the last data point (MAPE of 36.7%). As shown in Figure 6c, this consistency extends to the corresponding complex shear moduli. This performance suggests the empirical model's applicability across various UMR sizes and design philosophies.

For UMR‐L7.0P1.0, Figure 6d shows that Equation (6) accurately captures the step‐out frequency across the full range of tested viscoelastic conditions (MAPE of 9.8%). These results further validate the predictive capability and generalizability of the proposed dimensionless framework across varied UMR designs and actuation scenarios.

The empirical model was derived by fitting UMR performance data in gelatin phantoms with varying concentrations. Extrapolation of the model to other UMR designs requires a calibration measurement in a known environment to determine the calibration constant $C_{\mathrm{umr}}$. From this calibration measurement, the step‐out frequency across a range of gelatin concentrations can be accurately predicted. With the inclusion of $C_{\mathrm{umr}}$, the proposed model can be applied to various UMR design philosophies; the results demonstrate successful prediction for UMRs designed both for drilling and for swimming. While the experimental set covered only millimeter‐scale, closed‐body, screw‐shaped UMRs, the proposed modeling framework is expected to be applicable to alternative designs as well, such as hollow‐core UMRs or micro‐scale UMRs. The experimentally determined decay coefficient of 1.201 is likely sufficient for all millimeter‐scale UMR designs containing permanent magnets. For micro‐scale UMRs, magnetization is often achieved using ferromagnetic materials such as ${\mathrm{Fe}}_{3}O_{4}$ and nickel [36, 37]. These ferromagnetic materials are then magnetized by an external magnetic field, giving the micro‐scale UMR a magnetic dipole moment and introducing a relation between the magnetic field B and the UMR magnetic dipole m. Once this relation is incorporated into the proposed model, micro‐scale UMR performance could be estimated. The decay coefficient may need to be recalibrated to account for these fundamental differences in magnetic actuation and scaling behavior. Nevertheless, the proposed framework has been shown to predict the step‐out frequency of multiple screw‐shaped UMR designs with varying geometric and magnetic properties across soft tissues spanning a wide range of stiffnesses.

### 2.5 Model Performance in Resected Ex Vivo Brain Tissue

To evaluate how accurately the empirical model translates to real biological environments, the step‐out frequency of the microrobot UMR‐L7.0P1.0 was measured in resected ex vivo ovine brain tissue (Materials and Methods). The UMR‐L7.0P1.0 configuration was selected because it exhibits the highest predicted step‐out frequency across the investigated complex modulus range, Figure 7b. This makes it the most suitable test case for evaluating the predictive capability of the empirical model when transitioning from gelatin phantoms to heterogeneous biological tissue.

Prior to experimentation, the UMR was coated with a lipid‐based layer to reduce adhesion between the biological tissue and the robot surface, while simultaneously improving hemocompatibility and biocompatibility [31]. To enable prediction of the step‐out frequency in brain tissue based on gelatin calibration data, rheological measurements were performed on ovine brain samples and compared to those obtained from gelatin phantoms.

Rheological characterization of three independent ovine brain specimens revealed a strong dependence of the complex shear modulus on the applied shear rate (Figure 8a), in contrast to gelatin, where the complex modulus remained constant over the applied shear rates (Figure 6a). However, the range of complex moduli of the evaluated gelatin concentrations was comparable to that of the ovine brain tissue. Notably, large variations in magnitude of the complex modulus were observed between the three characterized brains, highlighting substantial inter‐sample heterogeneity of brain tissue. This heterogeneity arises because ex vivo brain tissue is highly susceptible to minor variations in preparation and deforms under its own weight, thereby altering its mechanical response [38, 39, 40].

**FIGURE 8:**
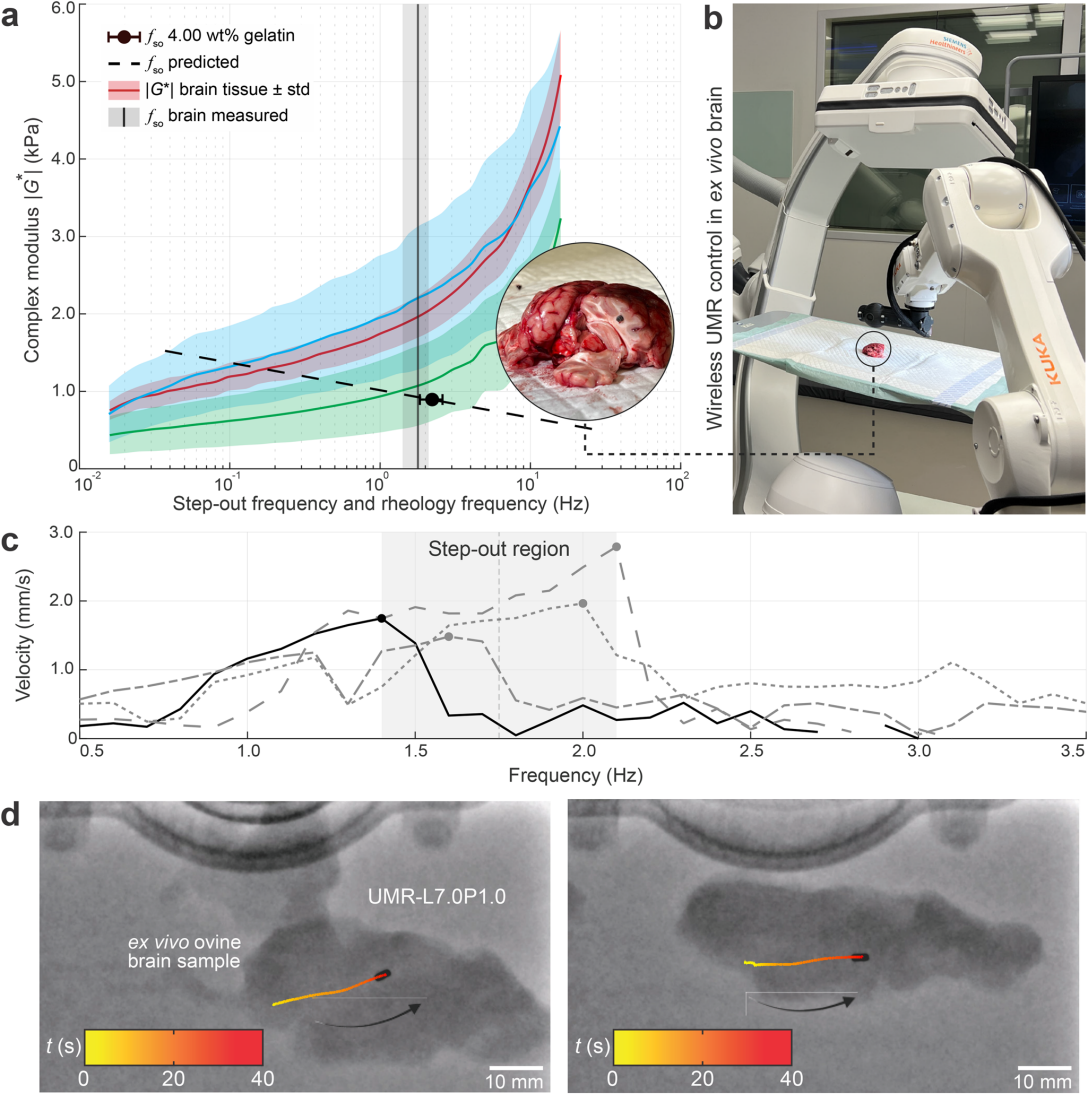
Estimation of the step‐out frequency and corresponding viscoelastic modulus of ex vivo ovine brain tissue using UMR‐L7.0P1.0, a drill‐type magnetic robot with a length of 7.0 mm and thread pitch of 1.0 mm. (a) Prediction of step‐out frequency based on the intersection between the complex modulus of gelatin and the corresponding complex modulus of ovine brain tissue, ranges from 0.12 Hz to 1.25 Hz step‐out frequency. The red, green, and blue solid lines are the mean of the complex moduli of the different ovine brain tissues, the shaded region illustrates the span of the measurements (*n*=6, 3, and 7 respectively). The black dashed line is the fitted line between the measured step‐out frequency in gelatin at known complex moduli. The gray shaded region illustrates our measured range of step‐out frequencies within the brain tissue. (b) The UMR is inserted into the ex vivo brain sample and its motion is visualized using X‐ray Fluoroscopy. (c) A frequency sweep was applied from 3.5 Hz to 0.5 Hz. At high frequencies almost no movement is seen, around 1.78 Hz the actuation frequency synchronizes with the robot and the robot achieves its maximum velocity (Movies S8–S11). (d) X‐ray Fluoroscopy images showing the rotating permanent magnet (top), ex vivo ovine brain tissue (gray region), and the embedded UMR (black). The UMRs trajectory is indicated for two trials as color‐scale lines.

Assuming that the dominant shear rate experienced by the surrounding medium is directly correlated with the rotational (angular) frequency of the UMR, the step‐out condition can be estimated by evaluating the complex modulus at the corresponding frequency. Accordingly, the complex modulus in Equation (6) can be expressed as a function of the step‐out frequency, $|G_{\mathrm{brain}}^{*}\left(f_{\mathrm{so}}\right)|$.

Using this assumption, the expected step‐out frequency in brain tissue was predicted by intersecting the empirical model of Equation (8), calibrated using a single reference point in 4 wt.% gelatin (black dashed line in Figure 8a), with the measured brain rheology data. Relating the UMR model curve to the mean trend of the three brain rheology datasets yields a predicted step‐out frequency range of approximately 0.12 Hz to 1.25 Hz. This wide range directly reflects the large variability in complex modulus observed across the tested brain samples.

The step‐out frequency of UMR‐L7.0P1.0 was measured experimentally ex vivo in resected ovine brain tissue (Figure 8b). A frequency sweep was applied using a rotating magnetic field, while the UMR position was continuously tracked via X‐ray Fluoroscopy images. Note that the frequency sweep was applied from 3.5 Hz down to 0.5 Hz, to prevent UMR displacement until the step‐out frequency was reached. In total, four trials were conducted using tissue from two separate ovine brains (Figure 8c). The corresponding translational trajectories of the UMR are shown in Figure 8d, from which the instantaneous velocities were extracted. A distinct and abrupt decrease in translational velocity at higher actuation frequencies indicates the step‐out behavior, which is summarized in Figure 8c based on the trajectory data of Figure 8d.

Across all trials, the measured step‐out frequencies ranged from 1.4 Hz to 2.1 Hz (Movies S8–S11), with a mean value of 1.78 Hz. Although the experimentally measured values are higher than the empirically predicted range of 0.12 Hz to1.25 Hz, the discrepancy is not large when considered in the context of the substantial variability in brain tissue rheology. The heterogeneity evident in Figure 8a naturally leads to a wide distribution of step‐out frequencies, which is further confirmed by the spread of experimentally measured values.

Several factors may contribute to the remaining differences between predicted and measured step‐out frequencies. First, surface friction between the UMR and the surrounding medium differs between gelatin and brain tissue [41]. While this effect is implicitly captured in the calibration constant $C_{\mathrm{UMR}}$, this constant was determined using gelatin phantoms and was not adjusted for brain tissue. Differences in surface adhesion alter how strongly the environment resists UMR rotation, thereby influencing the step‐out frequency. Because friction in gelatin is higher than in most biological tissues, including brain tissue, the model is likely to underestimate the step‐out frequency. Importantly, this surface‐friction‐related effect is expected to be largely consistent across UMR designs and could therefore be incorporated explicitly into future refinements of the empirical model.

Second, brain tissue is strongly heterogeneous, consisting of both gray and white matter. Due to the higher stiffness of white matter [42], the rheology of the brain tissue is likely shifted toward the stiffer white matter. During the step‐out frequency experiments, however, the UMR traversed regions containing both gray and white matter and therefore experienced a broader range of local mechanical environments than those represented by the bulk rheological measurement alone. As a result, the measured tissue modulus may overrepresent the influence of white matter, causing the model to overestimate the effective tissue resistance encountered by the UMR and consequently predict a lower step‐out frequency than observed experimentally.

Despite these sources of discrepancy, the empirical model successfully predicted the order of magnitude and practical operating range of the step‐out frequency at which the UMR was able to navigate through ex vivo brain tissue. This agreement supports the applicability of the gelatin‐based calibration approach for estimating UMR performance in complex, heterogeneous biological environments. Furthermore, a model that underestimates the step‐out frequency is preferable to one that overestimates it. Operating below the predicted step‐out frequency ensures that the UMR remains synchronously coupled to the rotating magnetic field, enabling stable and controllable navigation. In contrast, if the actual step‐out frequency is lower than predicted, the UMR may be actuated beyond its synchronization limit, resulting in loss of magnetic coupling, reduced propulsion efficiency, and uncontrolled oscillatory motion. Such behavior can compromise navigation accuracy and may increase the risk of mechanical damage to the surrounding tissue. Therefore, a conservative prediction of the step‐out frequency provides a safer operational margin for both experimental and future clinical applications.

### 2.6 Step‐Out Frequency Measurements in Perfused Ovine Brain

To further close the translational gap toward in vivo applications, experiments were conducted in a perfused ex vivo brain model, providing a more physiologically relevant environment while maintaining the experimental control required for systematic evaluation. As brain tissue characteristics change upon resection, a comparison to non‐resected brain tissue is necessary [39]. Comparing predictions from the ex vivo resected brain to the perfused ovine brain, clear differences in the experimental environment must be noted. During the rheological measurements in Figure 8, the brain was not perfused, did not contain blood, and was not pressurized, which alters the mechanical properties of the tissue. Consequently, the predictions are more representative of resected tissue conditions [38, 39, 40].

To evaluate the effect of blood perfusion, the step‐out frequency of UMR‐L7.0P1.0 was measured in a perfused ovine brain (Materials and Methods). Similar to the measurements in resected tissue, the UMR was coated prior to experimentation with a lipid‐based layer [31]. The applied frequency sweep of UMR‐L7.0P1.0 in the perfused ovine brain showed a clear difference in velocity as a function of actuation distance (Figure 9a). For each trial, the UMR was inserted into the brain and actuated using an RPM (Figure 9b). During the frequency sweep, the UMR was visualized using X‐ray fluoroscopy, from which translational trajectories were extracted (Figure 9c). After successful navigation within the ovine brain, a CBCT reconstruction was acquired, showing the location of the UMR within the tissue (Figure 9d).

**FIGURE 9:**
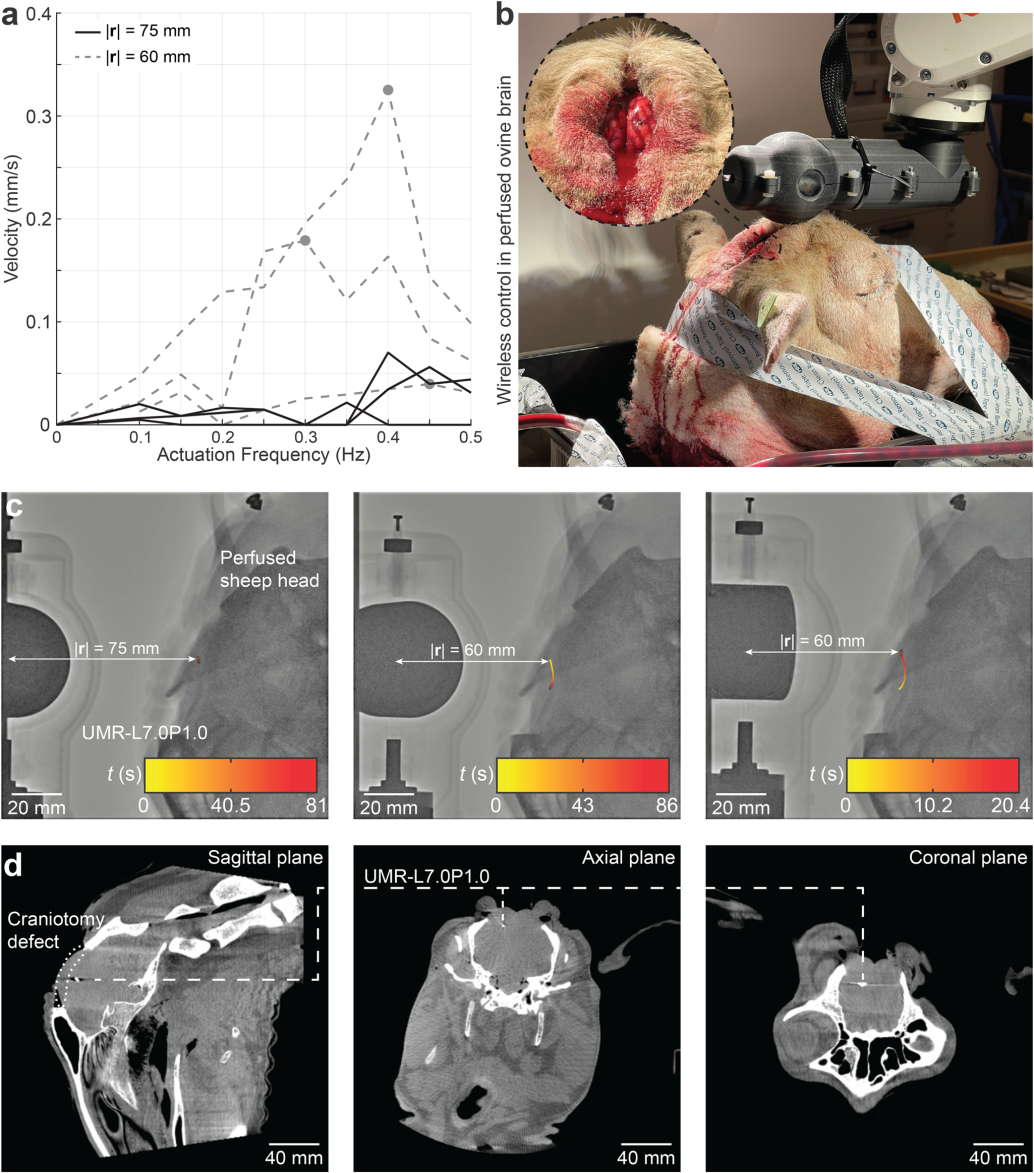
Measurement of the step‐out frequency of UMR‐L7.0P1.0, a drill‐type untethered magnetic robot (UMR) with a length of 7.0 mm and a thread pitch of 1.0 mm, in a perfused ovine brain. (a) Measured step‐out frequency of UMR‐L7.0P1.0 at actuator‐to‐robot gaps, |r|, of 75 mm and 60 mm. For an actuator gap of 60 mm, the measured step‐out frequency (highlighted points) was between 0.30 Hz and 0.45 Hz. At an actuator gap of 75 mm, only minimal movement was observed, preventing determination of the step‐out frequency. (b) Experimental setup. The sheep head was perfused through the carotid arteries, and a craniotomy was performed to expose the ovine brain. The UMR was inserted into the perfused brain tissue, and its motion was visualized using X‐ray fluoroscopy. (c) X‐ray fluoroscopy images showing both the rotating permanent magnet and the UMR. The UMR trajectory is indicated by a three‐color line scale. The UMR was navigated into the ovine brain tissue using actuation frequencies ranging from 0.5 Hz to 0.1 Hz at actuator‐to‐robot gaps of 75 mm (left) and 60 mm (middle), and subsequently navigated back to the insertion point at an actuation frequency of 1 Hz (right). (d) Sagittal, axial, and coronal views showing the position of the UMR within the brain tissue. The sagittal view also shows the craniotomy defect used for UMR insertion.

Comparing the navigation results at an actuator gap of 75 mm in perfused brain tissue (Figure 9) to those in resected brain tissue (Figure 8) shows that the motion of the UMR in the perfused brain tissue was highly limited, indicating actuation above the step‐out frequency. This reduction in performance can be attributed to increased pressure within the perfused brain tissue. In the perfused brain, blood perfusion pressurizes the tissue, increasing its effective stiffness and exerting additional normal forces on the UMR surface, thereby increasing surface friction and tissue resistance. Reducing the actuator gap to 60 mm enabled sufficient motion to determine the step‐out frequency. At this gap, the step‐out frequency was between 0.35 Hz and 0.45 Hz, highlighting the increased tissue resistance in perfused compared to resected brain tissue.

After successful navigation into the brain the UMR stayed in place (Figure 9d) without visual bleeding within the created channel. During retrieval of the UMR back to the insertion site the actuation frequency could be increased to 1 Hz, this increase can be attributed to the created channel of the UMR during insertion. The large increase in step‐out frequency during retrieval adds an inherent safety aspect to the use of UMRs within soft tissue. During retrieval the actuation frequency can be so much greater that the UMR will be unable to drill into the tissue, thereby forcing the UMR to navigate back through the original channel created during insertion.

Following the measured step‐out frequencies in both resected and perfused brain tissue, an estimate of the corresponding gelatin concentrations for control evaluation can be made. The measured step‐out frequencies were 1.78 Hz in resected brain tissue and below 0.1 Hz in perfused brain tissue. Solving Equation (6) for these step‐out frequencies yields complex moduli of 931 Pa and at least 1450 Pa for resected and perfused brain tissue, respectively. From Equation (1), it follows that gelatin concentrations of 4.1 wt.% and at least 4.9 wt.% can be used to create phantoms representative of resected and perfused brain tissue, respectively (Figure 5). These phantoms can be used to evaluate UMR motion control under realistic ex vivo conditions. This knowledge enables rapid prototyping of different UMR feedback controllers (Figure 4), thereby accelerating controller development.

## 3 Conclusions and Outlook

This study demonstrates a systematic approach for predicting the behavior of UMRs in soft viscoelastic materials. The presented empirical framework, based on a magnetic flux density, UMR magnetic moment, length, and mass, enables rapid estimation of UMR step‐out frequency across a range of soft media using a single calibration measurement in a reference material with a known complex modulus. The direct applicability of the model was validated by predicting UMR behavior ex vivo in resected ovine brain tissue. To date, predicting locomotion performance across multiple UMR designs and multiple tissue environments has remained largely inaccessible; the realization of such an empirical model therefore represents a major step toward the clinical deployment of UMRs. Following the proposed prediction approach reduces the need for studies directly in biological tissue, which are impractical due to limited availability, biological variability, ethical considerations, and tissue degradation over time. Consequently, such investigations must initially be conducted in well‐characterized tissue phantoms, with experimental workload increasing as environmental complexity rises. By establishing a quantitative relationship between tissue rheology and phantom composition, the proposed framework enables the design of physiologically relevant phantoms that reproduce the mechanical environment encountered in biological tissue. In this way, the framework provides a scalable, reproducible, and cost‐effective platform for large‐scale experimentation, controller development, and system optimization before progressing to increasingly realistic ex vivo and ultimately in vivo models. Moreover, the successful validation of the model in ex vivo ovine brain tissue demonstrates that the proposed approach extends beyond gelatin phantoms and can provide meaningful predictions in real biological tissues.

The presented model accurately captures the step‐out frequency of UMRs with varying sizes and design parameters across different gelatin phantoms, yielding a MAPE between 9.8% and 36.7%. This level of predictive accuracy enables rapid estimation of UMR step‐out frequency in soft tissues and substantially accelerates the development and optimization of task‐specific UMR geometries. Moreover, the step‐out frequency is not only a key factor of propulsion velocity, but is also closely linked to control stability. Consequently, the insights provided by our study support the development of real‐time feedback strategies and facilitate faster tuning of control parameters in closed‐loop UMR control systems.

Beyond performance prediction, the presented empirical framework provides a foundation for the rational design of magnetic microrobots intended for operation in delicate soft tissues, such as the brain. In envisioned translational scenarios, UMRs could be delivered endovascularly and subsequently traverse short distances into the parenchyma to enable precision biopsy or localized therapeutic delivery in deep‐seated gliomas or other lesions that are inaccessible to conventional neurosurgical approaches. More broadly, our model provides a generalizable foundation for optimizing microrobot geometry and actuation strategies in soft‐tissue environments, representing a key step toward future clinical translation.

## 4 Materials and Methods

### 4.1 Experimental Design

A systematic experimental protocol was implemented to evaluate the step‐out frequency of four uniquely designed UMRs across a range of viscoelastic environments. Each UMR, differing in geometry and magnetic moment, was actuated using a robotically controlled rotating permanent magnet. The step‐out frequency was identified by gradually decreasing the actuation frequency until the UMR starts to maintain synchronous rotation. Experiments were conducted in tunable gelatin‐based phantoms with controlled gelatin concentrations and ex vivo in perfused ovine brain tissue to represent physiologically relevant stiffness conditions. The complex shear modulus $|G^{*}|$ of each phantom was determined via rheological measurements, enabling dimensionless analysis of the relationship between UMR design, magnetic actuation, and tissue mechanics. These results were used to construct and validate a predictive model for step‐out behavior in soft biological media.

### 4.2 Experimental Setup and Actuation System

The magnetic actuation system consisted of a 6‐DOF industrial robotic manipulator that carried a cylindrical NdFeB permanent magnet (Figure 10a) (diameter = 45 mm, height = 30 mm, grade N45) (S‐45‐30‐N Supermagnete, Germany). The manipulator was placed above the UMR and the magnet was rotated, generating a rotating magnetic field along the long axis of the UMR. Frequencies were swept incrementally from 40 Hz down to 0.5 Hz, and step‐out was defined as the frequency beyond which the robot no longer exhibited asynchronous rotation or visible backward slipping (Movies S4–S7).

**FIGURE 10:**
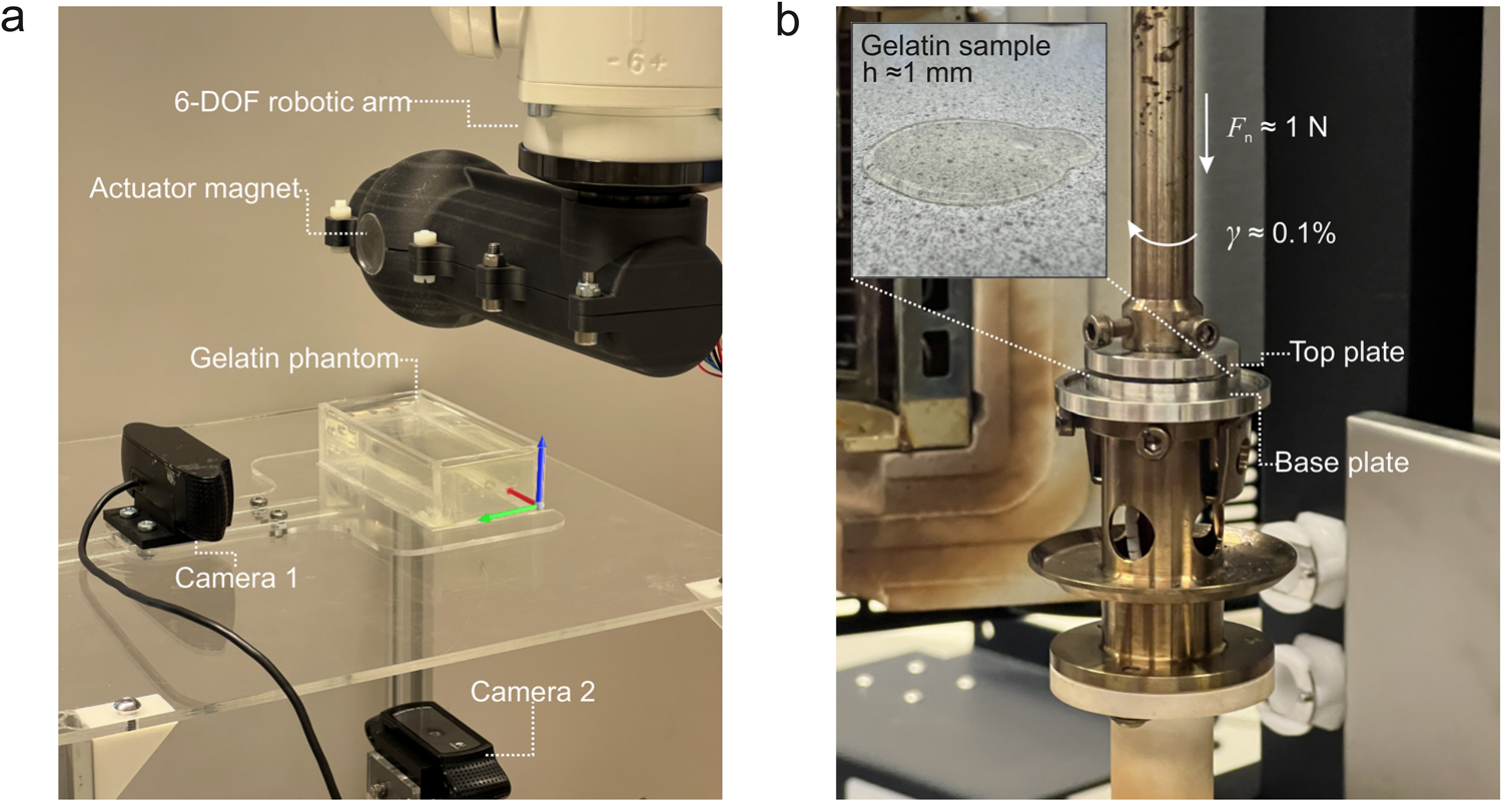
Untethered magnetic robot actuation setup and gelatin phantom characterization setup. (a) A rotating permanent magnet (RPM) is mounted to a motor whose position and orientation is controlled by a 6‐degree of Freedom (6‐DOF) robotic arm. Rotation of the RPM generates a rotating magnetic field within the gelatin phantom, actuating the untethered magnetic robot (UMR). The UMR's motion is recorded using two cameras. (b) A rheometer compresses a gelatin sample (25 mm in diameter, 1 mm thick; see inset) under an axial force of 1 N. A frequency sweep at 0.1 % strain is performed for each gelatin concentration, with a total of 3 samples per concentration.

### 4.3 Design and Fabrication of Untethered Magnetic Robots

To investigate how various designs of UMRs influence performance, four UMR prototypes were developed (Figure 2). Three robots were designed for soft‐tissue drilling (UMR‐L6.8P2.0, UMR‐L5.3P1.0, UMR‐L7.0P1.0) [24] (SI Appendix, Table S1), and one (UMR‐L6.4P1.8–6.0) for arterial swimming [43]. However, the latter also successfully drilled in gelatin‐based phantoms. The robots were designed using SolidWorks (Dassault Systémes, SolidWorks Corp. Inc., USA) to be printed in two halves. The central part of the robot was a cylinder. Inside the cylinder a small magnet was placed with magnetization value m. On the outside of the cylinder a thread was located allowing the UMR to drill in soft materials. All the drilling robots have a thread with constant pitch. The drilling UMRs possessed sharp, pointed ends designed specifically to facilitate penetration and minimize resistance within soft tissue. The swimming UMR had a variable pitch along its body and tip, along with larger fins at the tips to enhance propulsion efficiency within blood [20].

The UMRs were fabricated using Masked Stereolithography (MSLA) with an Elegoo Saturn 4 Ultra 16K 3D printer. Phrozen Aqua Grey 8K resin was selected as the structural material. Each UMR was printed in two distinct halves, with a layer thickness of 40 μm set in the ChiTuBox slicing software. This split parts approach allowed for the precise insertion of cylindrical magnets into the core structure and helped preserve the UMRs critical microfeatures (SI Appendix, Figure S1). After printing, a standard post processing protocol was followed: the halves were first cleaned in an isopropyl alcohol (IPA) ultrasonic bath for 10 min to remove residual resin, then exposed to UV light for 12 min to ensure complete polymerization and mechanical stability. Once the magnets were inserted into the core, the two halves were permanently bonded to form the complete UMR body. UMR‐L6.8P2.0 and UMR‐L7.0P1.0 contained a diametrically magnetized cylindrical magnet (1.2 × 3.0 mm, grade N35; HKCM Engineering, Germany), whereas UMR‐L5.3P1.0 and UMR‐L6.4P1.8–6.0 contained a diametrically magnetized cylindrical magnet (0.8 × 2.0 mm, grade N52; HKCM Engineering, Germany). Finally, the UMRs used for the ex vivo trials were coated with LipoCoat 4AC coating technology (LipoCoat BV, The Netherlands).

### 4.4 Untethered Magnetic Robot Closed‐Loop Control System

Closed‐loop control of the UMR was achieved by reconstructing the UMRs position and orientation, this was done by using a dual‐camera system, Figure 10a. After insertion of both the target and the UMR into a 4 wt.% gelatin phantom, a spline was drawn from the UMR toward the target as a reference path. The splines covered a range of about 100 mm in x‐direction, 25 mm in y‐direction and 14 mm in z‐direction. The UMR was actuated at 0.4 Hz, the actuator position was controlled using a 6‐degree of freedom robotic arm (KUKA KR10 R1100‐2, KUKA AG). The actuator magnet was kept directly above the UMR to ensure magnetic coupling as the UMR progressed along its path. Yawing motion of the UMR was achieved by yawing the actuator magnet, pitching motion of the UMR was achieved by slight lateral offsets between the UMR and actuator magnet. After each trial, the UMR was removed, the gelatin phantom was reset by melting and remolding.

### 4.5 Dimensional Analysis and Pi Group Formulation

When a UMR with magnetic dipole moment m (units: A·m 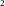) is placed in a magnetic field B (units: kg·A 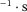 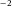), it experiences a magnetic torque:

(7) $$\tau_{f}=m\times B\;.$$

When driven by a rotating permanent magnet (RPM) with angular velocity $\omega_{f}$, a torque is generated around the principal axis of the UMR, $\tau_{f,p}$. This torque causes the UMR to spin with angular velocity $\omega_{u}$. In the synchronous regime, the RPM and UMR have the same angular velocity. Increasing the field rotation rate increases both $\omega_{u}$ and the viscous drag torque about the principal axis, $\tau_{d,p}$, since drag is linearly dependent on angular velocity:

(8) $$\tau_{d,p}=C_{D}\;\omega_{u}\;,$$

where $C_{D}$ is the viscous drag coefficient, dependent on both the UMR geometry and the surrounding medium. As long as $\tau_{f,p}>\tau_{d,p}$, the UMR remains synchronous with the magnetic field. Above a critical frequency, the step‐out frequency $f_{\mathrm{so}}$, the drag exceeds the field‐induced torque and the UMR falls out of synchrony, rotating more slowly than the applied field.

Predicting $f_{\mathrm{so}}$ analytically for arbitrary UMR designs in different media is challenging due to the many influencing variables, especially the complex viscous interactions incorporated in $C_{D}$. To reduce this complexity, the Buckingham Pi theorem can be applied [30]. For a problem with N variables of which K fundamental dimensions, the theorem states that the system can be expressed in terms of N−K dimensionless Pi groups. This allows the original variables to be expressed using fewer, independent dimensionless Pi groups.

In this case, we use four fundamental dimensions: mass [kg], length [m], time [s], and electric current [A]. So K=4. The base variables, chosen to span the four fundamental dimensions, are given in Table 1.

**TABLE 1:** Base variables used to create dimensionless Pi groups.

Base variable	Symbol	Unit
UMR length	L	(m)
UMR mass	m	(kg)
UMR dipole moment	|m|	(A·m 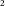 )
Magnetic flux density	|B|	(kg·A 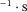 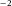 )

Any material or UMR property influencing the step‐out frequency $f_{\mathrm{so}}$ (unit: $s^{-1}$) can be non‐dimensionalised using these four base variables. To capture both the viscous and elastic behavior it was chosen to use the complex shear modulus $|G^{*}|$ (units: kg $\cdot m^{-1}\cdot$ $s^{-2}$) to describe the material. The two dimensionless Pi groups for $f_{\mathrm{so}}$ and $|G^{*}|$ are then:

(9) $$\Pi_{1}=f_{\mathrm{so}}\sqrt{\frac{L^{2}m}{\left|m||B\right|}}\;\;\;\text{and}\;\;\;\Pi_{2}=\left|G^{*}\right|\frac{L^{3}}{\left|m||B\right|}\;,$$

where $\Pi_{1}$ represents the dimensionless form of the step‐out frequency and $\Pi_{2}$ the dimensionless form of the complex shear modulus. Besides these Pi groups, there are additional dimensionless groups related to the design of the UMR, which will be called $\Pi_{\text{UMR}}$. $\Pi_{\text{UMR}}$ covers, for example, the UMR diameter or the number of fins. Due to the complex geometry, it is difficult to identify all relevant Pi groups. However, since these parameters are fixed for a given UMR, $\Pi_{\text{UMR}}$ should not change across different media (assuming |B| remains constant for one UMR).

The Buckingham Pi theorem implies that

(10) $$\Pi_{1}=F\left(\Pi_{2},\Pi_{\mathrm{UMR}}\right)\;.$$

Here we propose the separable form

(11) $$\Pi_{1}=F_{\mathrm{mat}}\left(\Pi_{2}\right)\;C_{\mathrm{UMR}}\;,$$

where $C_{\text{UMR}}$ is a constant depending on the UMR design and magnetic field. $F_{\mathrm{mat}}\left(\Pi_{2}\right)$ captures the material response universal for all UMRs. By systematically varying $\Pi_{2}$ (e.g., by changing the complex shear modulus $|G^{*}|$ of the medium) while keeping $C_{\text{UMR}}$ constant, the function $F_{\text{mat}}\left(\Pi_{2}\right)$ can be empirically determined.

Because $C_{\text{UMR}}$ is a constant depending on the UMR design, and $F_{\text{mat}}\left(\Pi_{2}\right)$ is universal for different UMR designs, one can easily determine $C_{\text{UMR}}$ by measuring $\Pi_{1}$ (the step‐out frequency) in a single medium with a known complex modulus. Once $C_{\text{UMR}}$ is determined, the model allows prediction of the step‐out frequency for that UMR in other media by substituting the material properties in $\Pi_{2}$.

### 4.6 In Vitro Phantom Media Preparation and Rheological Characterization

Viscoelastic phantoms were prepared by dissolving gelatin powder (Dr. Oetker Professional, 250 bloom) in water at 50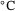. The resulting solution was poured into rectangular molds (10 × 5 × 3 cm) and allowed to cool and solidify at 4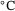. Prior to testing, the phantoms were removed from the refrigerator and allowed to reach room temperature.

To characterize their viscoelastic properties, rheological measurements were performed on a Discovery HR‐20 rheometer (TA Instruments, USA) equipped with 25mm parallel plates (Figure 10b). A frequency sweep (0.1 % strain, 0.1rad/sto100rad/s, 10 points per decade) was applied to 1mm‐thick samples spanning gelatin concentrations from 3 wt.% to 7 wt.%. To prevent slip between the sample and the parallel plates a small strain was used and also a small normal force (1N) was applied by the parallel plates.

The relationship between gelatin concentration and complex modulus (Figure 5(d)) was compared to that reported by the rheometer's manufacturer, TA Instruments [32]. Here the following relation was found:

(12) $$|G^{*}|=62.9c_{{\mathrm{gel}}_{\mathrm{TA}}}^{2}-123.8c_{{\mathrm{gel}}_{\mathrm{TA}}}+45.8.$$

To correct for the difference in bloom value (B), $B_{\mathrm{TA}}$ = 175 from the gelatin used by TA Instruments to the gelatin used in this study with bloom value of B = 250, a compensation was applied as proposed by Francis [33],

(13) $$c_{\mathrm{gel}}=c_{{\mathrm{gel}}_{\mathrm{TA}}}\frac{\sqrt{B_{\mathrm{TA}}}}{\sqrt{B}}.$$

Resulting in the relation between gelatin concentration and complex modulus as described in Equation (1).

### 4.7 Ex Vivo Cerebral Tissue Model

To evaluate microrobot performance in a physiologically relevant environment, we conducted experiments using an ex vivo ovine brain model. Animal tissue was obtained from sheep euthanized as part of an unrelated animal study, approved by Central Authority for Scientific Procedures on Animals (CCD AVD10300202226109). No animals were euthanized or subjected to experimental procedures specifically for the purposes of this study. Sheep brains were extracted and prepared within one hour after death to preserve anatomical and mechanical integrity.

In the first set of experiments (Figure 3), the brain remained in situ within the skull, and the common carotid artery was cannulated. To prevent blood coagulation and maintain tissue hydration during overnight storage, the sheep head was perfused with saline. Prior to experimentation, the saline was replaced with sheep blood using a peristaltic pump. The UMR was then inserted into the brain through a small cranial incision, and the rotation frequency of the external magnetic field was gradually reduced until smooth locomotion was achieved.

In subsequent experiments (Figure 8), the brain was removed from the skull to allow for more controlled testing conditions. The samples were stored on ice overnight and allowed to reach room temperature prior to use. Step‐out frequencies were determined using the same protocol as in the gelatin phantoms. Rotation frequencies of the external magnetic field were gradually decreased until the step‐out threshold was reached, as identified through visual inspection and video tracking (Movies S8–S11). Forward velocities were quantified by tracking the UMR in X‐ray Fluoroscopy recordings.

Due to the inherent variability in tissue structure in the brain, tests were repeated in multiple cortical regions across different samples. Averaged step‐out frequencies were then compared with the predictions of the empirical model derived from gelatin‐based phantoms. This approach enabled external validation of the model and confirmed its applicability to brain tissue.

Characterization of the resected ovine brain tissue was performed using a AR2000 rheometer (TA Instruments, USA). To minimize rheological errors in the brain tissue, the location and orientation of the sample have been carefully considered [38]. The cerebrum was cut into 2–3 mm‐thick slices along the sagittal plane, perpendicular to the UMR propulsion direction. These slices of tissue were cut down to the size of the rheometer 8 mm plate using a 8 mm hole punch. A nominal force of about 0.01 N was applied and frequency sweeps (1% strain, 0.1 rad/s to 100 rad/s, 10 points per decade) were performed on the samples.

### 4.8 Perfused Ovine Brain Tissue

To further simulate in vivo conditions, experiments were conducted on a perfused ex vivo ovine brain. After death was confirmed, blood was collected via cardiac puncture. Following cannulation of both carotid arteries, the sheep head was removed from the body. Subsequently, the blood vessels were flushed with saline to prevent coagulation. A craniotomy was performed to provide direct access to the ovine brain, enabling multiple trials on the same tissue. The sheep head was stored overnight on ice and allowed to reach room temperature before use. Perfusion of the ovine brain was achieved by pumping blood through the carotid arteries. The flow rate through the carotids was approximately 80 mL/min, and the blood was heated to 37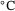 to maintain physiologically relevant tissue conditions. During the experiments, blood draining from the head was collected in a reservoir and continuously recirculated back to the carotid arteries using a pump. The craniotomy was then reopened, and a UMR was inserted into the brain tissue. After insertion, the actuator magnet was positioned directly above the UMR at the desired distance. The actuator‐to‐UMR gap was validated using an X‐ray scan. Once positioned, the actuator magnet was rotated with a frequency sweep from 0.5 Hz down to 0.1 Hz in steps of 0.05 Hz. At each frequency, the actuator was allowed to complete two full rotations. During actuation, the UMR position was recorded using X‐ray fluoroscopy. The UMR displacement was quantified from the resulting fluoroscopy videos. After navigation within the ovine brain, a CT scan was acquired for reconstruction. Removal of the UMR was achieved by reversing the rotational direction of the actuator, which was then spun at 1 Hz.

## Author Contributions

L‐J.W.L., T.J.vd B., S.Y.K., and R.L. performed experiments; L‐J.W.L., S.Y.K., and S.S.C. designed the untethered robots; T.J.vd B. and S.Y.K. created the empirical model; L‐J.W.L., T.J.vd B., S.Y.K., A.K, R.M.L.M.L., L.S.J., D.W., H.R.L, and I.S.M.K. analyzed experimental data; D.W. and P.J. ensured biocompatibility of the untethered robots; D.F.R., D.B.A., U.S., O.S., G.D., P.J., J.J.F., S.S., J.F.W.N., M.C.W., and I.S.M.K. facilitated the acquisition of financial support; L‐J.W.L., T.J.vd B., S.Y.K., S.S.C., R.M.L.M.L., and I.S.M.K participated in drafting the paper and revising it critically. All authors discussed the results and contributed to the final manuscript.

## Conflicts of Interest

P.J. is co‐founder of LipoCoat B.V and the other authors have no conflict of interest to declare.

## Supporting information




**Supporting File 1**: advs77011‐sup‐0001‐SuppMat.pdf.

**Supporting File 2**: advs77011‐sup‐0002‐MovieS1.mp4.

**Supporting File 3**: advs77011‐sup‐0003‐MovieS2.mp4.

**Supporting File 4**: advs77011‐sup‐0004‐MovieS3.mp4.

**Supporting File 5**: advs77011‐sup‐0005‐MovieS4.mp4.

**Supporting File 6**: advs77011‐sup‐0006‐MovieS5.mp4.

**Supporting File 7**: advs77011‐sup‐0007‐MovieS6.mp4.

**Supporting File 8**: advs77011‐sup‐0008‐MovieS7.mp4.

**Supporting File 9**: advs77011‐sup‐0009‐MovieS8.mp4.

**Supporting File 10**: advs77011‐sup‐0010‐MovieS9.mp4.

**Supporting File 11**: advs77011‐sup‐0011‐MovieS10.mp4.

**Supporting File 12**: advs77011‐sup‐0012‐MovieS11.mp4.

## Data Availability

The data that support the findings of this study are available from the corresponding author upon reasonable request.
