# Supplementary material for: Performance Estimation and Ex Vivo Validation of Untethered Magnetic Robots in Soft Tissue
Source: Adv Sci (Weinh). 2026 Aug 14;13(61):e77011. doi: 10.1002/advs.77011 (PMC13474191; doi:10.1002/advs.77011)
Supplement: Supplementary file 1 [file ADVS-13-e77011-s006.pdf]

Supporting Information for

Performance estimation and *ex vivo* validation of untethered magnetic robots in soft tissue

Leendert-Jan W. Ligtenberg, Thijs J. van der Burg , Stijn Y. Kolkman, Santiago Saavedra Castano, David Fernandez Rivas, Anke Klingner, Roger Lomme, Lucas S. Joziasse, Dorothee Wasserberg, H. Remco Liefers, Doron Ben Ami, Udi Sadeh, Oded Shoseyov, Giulio Dagnino, Pascal Jonkheijm, Jurgen J. Fütterer, Stefano S. Stramigioli, J. Frank W. Nijsen, Michiel C. Warlé, Islam S. M. Khalil

To whom correspondence should be addressed. Email: l.w.ligtenberg@utwente.nl, michiel.warle@radboudumc.nl, i.s.m.khalil@utwente.nl

This PDF file includes:

Supporting text
Fig. S1
Table S1
Legends for Movies S1 to S11
SI References

Other supporting materials for this manuscript include the following:

Movies S1 to S11

Supporting Information Text

Supplementary Methods

Robot design and manufacturing. The designs of the untethered magnetic robots UMRs were based on a simple screw design as this shape has shown to be effective in drilling soft tissue environments (1–3). The length and width of the UMR was dependent on the size of the internal magnet.

The geometry of the UMRs was designed using a combination of MATLAB and SOLIDWORKS. The design of the robot, including the design parameters can be seen in Fig.S1. The design used parameters are shown in Tab. S1. The body shape was first generated in MATLAB and subsequently refined in SOLIDWORKS for 3D printing. In MATLAB, the robot surface was defined using a polar parameterization (θ, ζ), where θ is the azimuthal angle around the central axis, between 0 and 2π, and ζ the axial coordinate along the UMR length, between 0 and the half UMR length $L_{\text{body}}/2+L_{\text{tip}}$. Only 1 half of the UMR was modeled due to the symmetry in its shape.

A radius profile $A(\zeta)$ describes the main body shape; a cylindrical section of length $L_{\text{body}}/2$ that transitions into a conical tip of length L_{tip}. The maximum body radius r_{body} is defined as the radius of the magnet r_{magnet} and radius of the UMR wall thickness r_{wall} added together, $r_{\text{body}} = r_{\text{magnet}} + r_{\text{wall}}$. To introduce helical fins with height h_{fin}, a periodic modulation function ρ is applied to the radius

$$\rho(\theta) = 1 + \frac{h_{\text{fin}}}{r_{\text{body}}} f(n_{\text{fin}}\theta),$$

where $f(\cdot)$ is a periodic square wave between 0 and 1, with a duty cycle of $\frac{w_{\text{fin}}\, n_{\text{fin}}}{p}$. Here p is the helical pitch of the UMR fin, w_{fin} is the thickness of the fin and n_{fin} is the number of fins the UMR has.

The resulting parametrized surface is calculated using $S(\theta, \zeta) = A(\zeta)\rho(\theta)$, where $S(\theta, \zeta)$ describes the surface of the UMR in polar coordinates. $A(\zeta)$ represents the base cylindrical body along the longitudinal axis, while $\rho(\theta)$ defines the fin shape that is periodically modulated around it.

$$A(\zeta) = \begin{cases} r_{\text{body}} & \text{if } \zeta \leq \frac{L_{\text{body}}}{2} \\ r_{\text{body}} \left(1 - \frac{\zeta - \frac{L_{\text{body}}}{2}}{L_{\text{tip}}}\right) & \text{if } \frac{L_{\text{body}}}{2} < \zeta \leq \frac{L_{\text{body}}}{2} + L_{\text{tip}} \end{cases}$$

The surface is then mapped to Cartesian coordinates

$$X = A(\zeta)\,\rho(\theta)\,\cos\left(\frac{2\pi}{p}\zeta + \theta\right),$$
$$Y = A(\zeta)\,\rho(\theta)\,\sin\left(\frac{2\pi}{p}\zeta + \theta\right),$$
$$Z = \zeta,$$

where p is the helical pitch. The resulting mesh was exported as an STL file for further processing. In SOLIDWORKS, the STL mesh was converted to a solid model suitable for printing. A planar surface was generated to close the base of the body, after which the surfaces were knitted and converted to a solid. Finally, an extruded cut was applied to create the cylindrical cavity for the embedded permanent magnet.

A UMR was printed in two halves using Masked Stereolithography (Phrozen Sonic Mini 4K, MSLA). The halves were printed pointing upwards as shown in Fig. S1(b) with the minimum amount of support as possible. After printing the a magnet was inserted in between the 2 halves of the UMR, resulting in a UMR as shown in as shown in Fig. S1(c). The resulting UMR length was measured and used as an input to the empirical model.

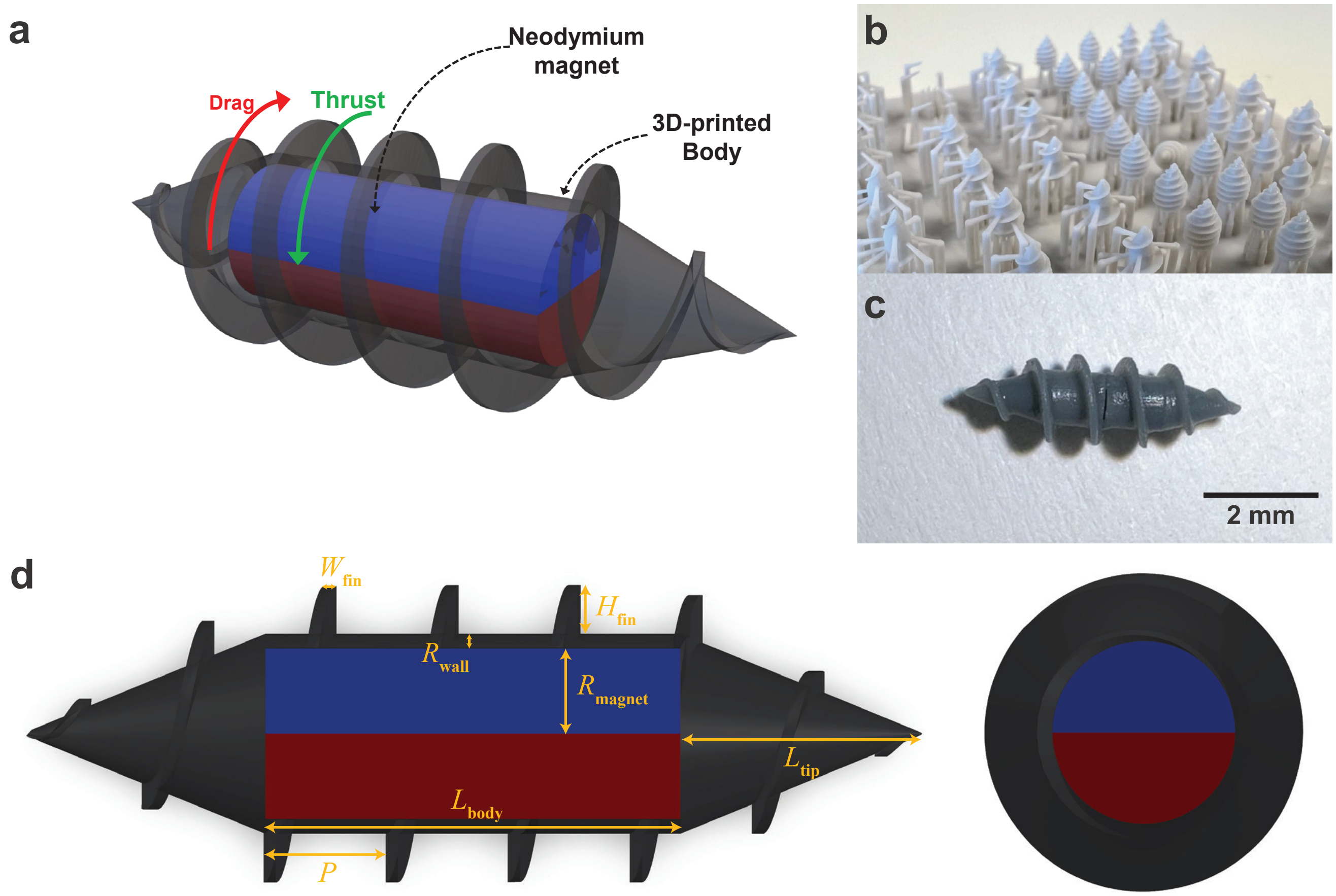

Fig. S1. Design and fabrication of the untethered magnetic robot (UMR). (a) Schematic of the UMR, consisting of a 3D-printed body housing a cylindrical neodymium magnet. Under an applied magnetic field, the UMR experiences thrust, while the surrounding medium exerts a drag. (b) The body of the robot is printed in two halves using Masked Stereolithography (Phrozen Sonic Mini 4K, MSLA). (c) Example of an assembled UMR (length 5.3 mm), fabricated by 3D-printing two helical segments and bonding them together with the magnet enclosed. (d) Cross-sectional views of the UMR CAD model, a diametrically magnetized cylindrical magnet is embedded inside a 3D-printed body.

Table S1. UMR design parameters with the corresponding value for each drilling UMR type

Name	Symbol	Units	Description	UMR-L6.8P2.0	UMR-L5.3P1.0	UMR-L7.0P1.0
Magnet radius	r_{magnet}	mm	Radius of the used magnet including a small printing tolerance depending on printer quality.	0.35	0.25	0.35
Wall thickness	r_{wall}	mm	Radial wall around the magnet; $r_{\text{body}} = r_{\text{magnet}} + r_{\text{wall}}$.	0.12	0.12	0.12
Body length	L_{body}	mm	Length of the cylindrical section (the script models half: $L_{\text{body}}/2$).	3.4	2.4	3.4
Tip length	L_{tip}	mm	Length of the conical tip.	2	2	2
Pitch	p	mm/rev	Axial advance per revolution; sets total turns.	2	1	1
Number of fins	n_{fin}	—	Number of equally spaced helical fins.	1	1	1
Fin height	h_{fin}	mm	Radial height of the fin.	0.4	0.4	0.4
Fin thickness	w_{fin}	mm	Fin thickness.	0.1	0.1	0.1

Movie S1. A drill-type untethered magnetic robot (UMR) with a length of 7.0 mm and thread pitch of 1 mm (UMR-L7.0P1.0) is propelled through an ovine brain using external magnetic fields. The UMR is navigating away from the insertion site, forming a new path.

Movie S2. A drill-type untethered magnetic robot (UMR) with a length of 7.0 mm and thread pitch of 1 mm (UMR-L7.0P1.0) is propelled through an ovine brain using external magnetic fields. The UMR is retracing the original path back to the insertion site.

Movie S3. Closed-loop control of an untethered magnetic robot (UMR) UMR-L7.0P1.0 in a 4 wt% gelatin phantom. The UMR was steered using externally applied rotating magnetic fields. The UMR trajectory was from its insertion (left) towards its target (right), the target was reached after 500 seconds.

Movie S4. Step-out measurement of an untethered magnetic robot (UMR) with a length of 6.8 mm and thread pitch of 2 mm (UMR-L6.8P2.0) in a 4 wt% gelatin phantom. Top left shows the rotational frequency at which the actuator magnet spins about its axis, red text indicates above step-out and green text indicates below step-out. Step-out frequency for this trial is 2.3 Hz

Movie S5. Step-out measurement of an untethered magnetic robot (UMR) with a length of 5.3 mm and thread pitch of 1 mm (UMR-L5.3P1.0) in a 4 wt% gelatin phantom. Top left shows the rotational frequency at which the actuator magnet spins about its axis, red text indicates above step-out and green text indicates below step-out. Step-out frequency for this trial is 2.3 Hz

Movie S6. Step-out measurement of an untethered magnetic robot (UMR) with a length of 6.4 mm and thread pitch of 1.8 mm and 6.0 mm (UMR-L6.4P1.8-6.0) in a 4 wt% gelatin phantom. Top left shows the rotational frequency at which the actuator magnet spins about its axis, red text indicates above step-out and green text indicates below step-out. Step-out frequency for this trial is 3.9 Hz

Movie S7. Step-out measurement of an untethered magnetic robot (UMR) with a length of 7.0 mm and thread pitch of 1 mm (UMR-L7.0P1.0) in a 4 wt% gelatin phantom. Top left shows the rotational frequency at which the actuator magnet spins about its axis, red text indicates above step-out and green text indicates below step-out. Step-out frequency for this trial is 4.0 Hz

Movie S8. Step-out measurement of an untethered magnetic robot (UMR) with a length of 7.0 mm and thread pitch of 1 mm (UMR-L7.0P1.0) in a resected ovine brain. Top left shows the rotational frequency at which the actuator magnet spins about its axis, red text indicates above step-out and green text indicates below step-out. Step-out frequency for this trial is 1.4 Hz

Movie S9. Step-out measurement of an untethered magnetic robot (UMR) with a length of 7.0 mm and thread pitch of 1 mm (UMR-L7.0P1.0) in a resected ovine brain. Top left shows the rotational frequency at which the actuator magnet spins about its axis, red text indicates above step-out and green text indicates below step-out. Step-out frequency for this trial is 2.1 Hz

Movie S10. Step-out measurement of an untethered magnetic robot (UMR) with a length of 7.0 mm and thread pitch of 1 mm (UMR-L7.0P1.0) in a resected ovine brain. Top left shows the rotational frequency at which the actuator magnet spins about its axis, red text indicates above step-out and green text indicates below step-out. Step-out frequency for this trial is 2.0 Hz

Movie S11. Step-out measurement of an untethered magnetic robot (UMR) with a length of 7.0 mm and thread pitch of 1 mm (UMR-L7.0P1.0) in a resected ovine brain. Top left shows the rotational frequency at which the actuator magnet spins about its axis, red text indicates above step-out and green text indicates below step-out. Step-out frequency for this trial is 1.6 Hz

References

1. M Sendoh, K Ishiyama, KI Arai, Direction and individual control of magnetic micromachine. *IEEE Transactions on Magn.* **38**, 3356–3358 (2002).
2. AW Mahoney, ND Nelson, EM Parsons, JJ Abbott, Non-ideal behaviors of magnetically driven screws in soft tissue in *2012 IEEE/RSJ International Conference on Intelligent Robots and Systems.* pp. 3559–3564 (2012).
3. ND Nelson, J Delacenserie, JJ Abbott, An empirical study of the role of magnetic, geometric, and tissue properties on the turning radius of magnetically driven screws in *2013 IEEE International Conference on Robotics and Automation.* pp. 5372–5377 (2013).
